# A simple and rapid pipeline for identification of receptor-binding sites on the surface proteins of pathogens

**DOI:** 10.1038/s41598-020-58305-y

**Published:** 2020-01-24

**Authors:** Patrícia Mertinková, Amod Kulkarni, Evelína Káňová, Katarína Bhide, Zuzana Tkáčová, Mangesh Bhide

**Affiliations:** 1 Laboratory of Biomedical Microbiology and Immunology, The University of Veterinary Medicine and Pharmacy, Komenského 73, 04001 Košice, Slovakia; 2 Institute of Neuroimmunology of Slovak Academy of Sciences, 84510 Bratislava, Slovakia

**Keywords:** Proteins, Proteomic analysis

## Abstract

Ligand-receptor interactions play a crucial role in the plethora of biological processes. Several methods have been established to reveal ligand-receptor interface, however, the majority of methods are time-consuming, laborious and expensive. Here we present a straightforward and simple pipeline to identify putative receptor-binding sites on the pathogen ligands. Two model ligands (bait proteins), domain III of protein E of West Nile virus and NadA of *Neisseria meningitidis*, were incubated with the proteins of human brain microvascular endothelial cells immobilized on nitrocellulose or PVDF membrane, the complex was trypsinized on-membrane, bound peptides of the bait proteins were recovered and detected on MALDI-TOF. Two peptides of DIII (~916 Da and ~2003 Da) and four peptides of NadA (~1453 Da, ~1810 Da, ~2051 Da and ~2433 Da) were identified as plausible receptor-binders. Further, binding of the identified peptides to the proteins of endothelial cells was corroborated using biotinylated synthetic analogues in ELISA and immunocytochemistry. Experimental pipeline presented here can be upscaled easily to map receptor-binding sites on several ligands simultaneously. The approach is rapid, cost-effective and less laborious. The proposed experimental pipeline could be a simpler alternative or complementary method to the existing techniques used to reveal amino-acids involved in the ligand-receptor interface.

## Introduction

Ligand-receptor interactions (LRIs) are the hallmark of the plethora of physiological process and host-pathogen crosstalk. Several methods are developed to investigate the amino acid residues involved in the ligand-receptor interface, e.g. yeast two-hybrid system^1–3^, mutational analysis^4–6^, protein microarrays^7,8^, X-ray crystallography^9–11^, and mass spectrometry (MS)^12^.

In this report, we aimed to combine MS and limited proteolysis (LP) of the ligand-receptor complex to identify amino acid residues plausibly involved in the ligand-receptor interface. So far, LP has been used in the studies to reveal protein structure^13–15^, protein domains^16,17^, binding sites^18–20^, chemical modifications^21^ and to drug targets^22,23^. Moreover, the protein conformational features deduced from LP experiments correlate with those derived from NMR spectroscopy, X-ray crystallography or circular dichroism^24–26^. Proteolysis of the native protein is restricted to the residues available on the surface, while in case of protein complexes, the surface residues involved in the ligand-receptor interface are buried and remain inaccessible to the proteases. This can be exploited to identify the amino acid stretch involved in the ligand-receptor interface. Several proteases have been applied successfully (e.g. chymotrypsin, LysC, ArgC, GluC, AspN, etc.)^21,27–29^ to digest the proteins for further applications like MS, however, trypsin remains commonly used protease.

Limited tryptic digestion was used before to reveal the amino acid sequence of the epitope within the antibody-antigen immune complex^30^, wherein monoclonal antibody was immobilized on tresyl-activated sepharose followed by incubation with antigen, LP, and identification of peptides with Cf plasma desorption MS^30^. The technique can easily be exploited for exposing amino acids involved in the LRIs. However, sepharose being a porous material mediates nonspecific binding of protein molecules. Alternatively, polystyrene in which amine group is loaded for further modification was employed for protein immobilization. Nitrocellulose (NC) and polyvinylidene difluoride (PVDF) membranes are commonly used matrices in the proteomic laboratories with inherent protein binding ability. These membranes can also be used for LP of the protein complexes to identify the interface of LRIs.

Implementation of the matrix-assisted laser desorption/ionization (MALDI) for MS has revolutionized the field of protein identification. So far, several methods of protein desalting, prerequisite for the MS, have been established. Among them, on-membrane (PVDF or NC) protein washing and desalting techniques are rapid and low-cost^31,32^. In these studies, after on-membrane digestion, the NC membrane was dissolved in MALDI matrix solution followed by MS analysis. However, the presence of the NC membrane along with the peptides hindered the performance of MS. To overcome this drawback, membrane was dissolved in organic solvents (like acetone, acetonitrile, etc.) and peptides were precipitated^33^. Similarly, PDVF membranes are also applicable to on-membrane protein digestion^34^.

Here, a rapid and simple methodological approach to map plausible protein-binding sites is presented, which combines tryptic LP of the ligand-receptor complex followed by recovery of the interacting peptide of the ligand and its identification on MALDI-TOF. Two model ligands namely protein E of West Nile virus (domain III, DIII) and neisserial adhesin A of *Neisseria meningitidis* (NadA, globular domain with the first coiled-coil structure) were used. Interaction of both proteins with the host cell receptors was previously reported^35–39^. Moreover, both proteins play an important role in the pathogenesis^40,41^ and are being explored as possible therapeutic targets^42–44^. We believe that the presented pipeline could help researchers to identify plausible receptor-binding sites on the protein ligands within the short-time, and with less labour and cost.

## Results

### Binding of recombinant DIII (rDIII) and NadA (rNadA) to the proteins of human brain microvascular endothelial cells (hBMECs)

**Figure 1:**
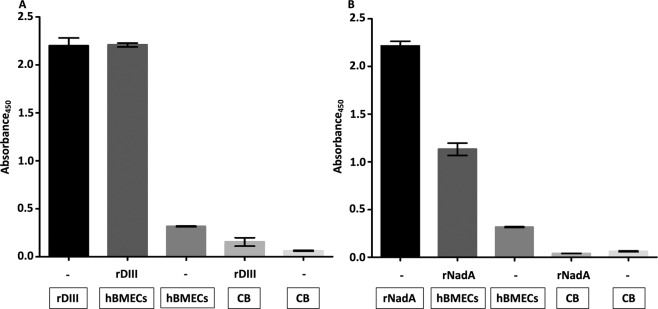
Assessment of interaction between recombinant ligands (rDIII and rNadA) and protein extract of hBMECs using semi-quantitative ELISA. A – rDIII; B - rNadA. Interaction was detected with HisProbe-HRP conjugate. Framed reagents were coated into microtiter wells. Data present means of triplicates with ± S.D. CB – coating buffer; hBMECs – protein extract of human brain microvascular endothelial cells; rDIII – recombinant DIII; rNadA – recombinant NadA. Please note that all wells were blocked with blocking buffer after overnight coating. Interaction was detected with HisProbe-HRP conjugate.

**Figure 2:**
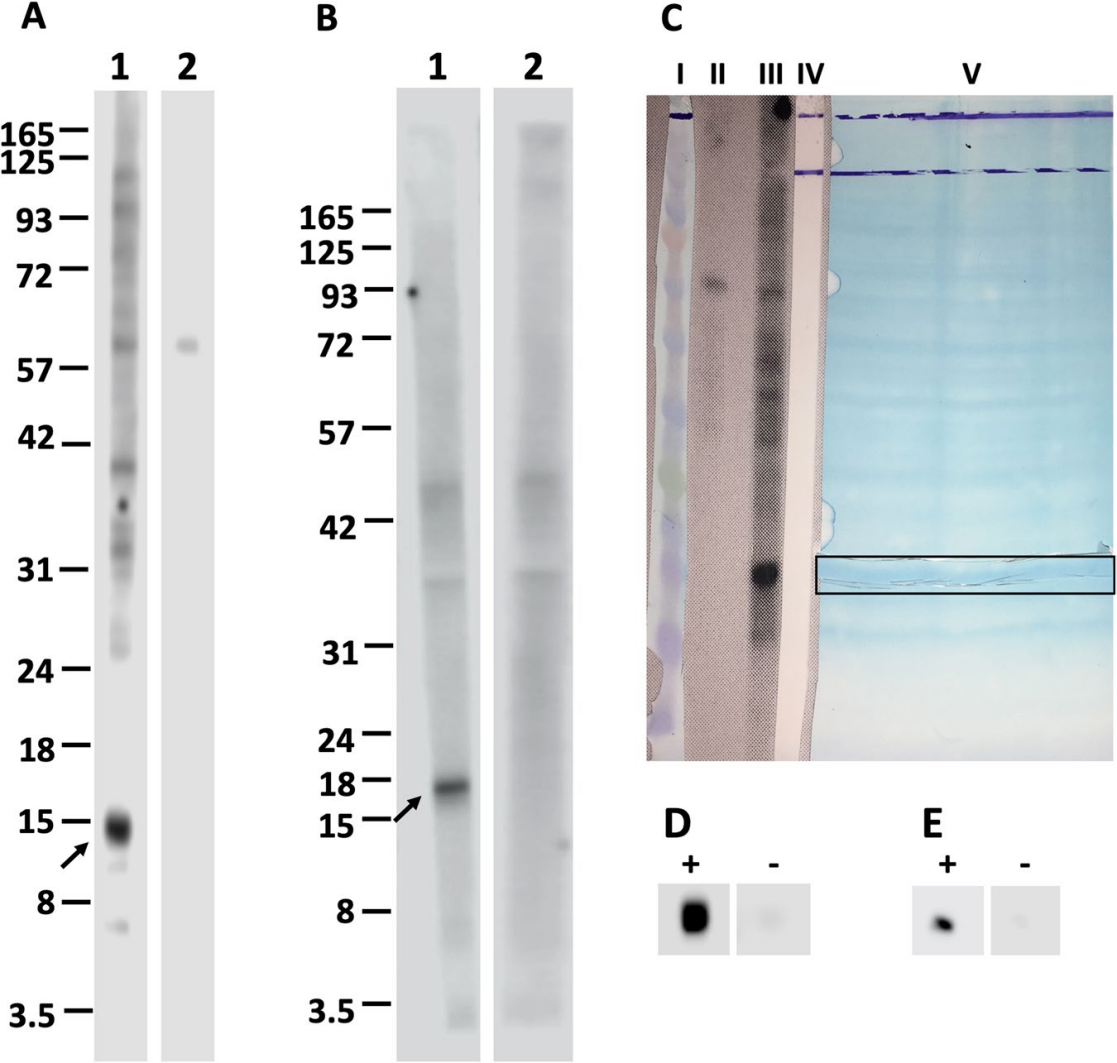
Confirmation of interaction between recombinant ligands and proteins of hBMECs using Western blotting. Nitrocellulose membrane strips with transblotted proteins of hBMECs were incubated either with recombinant ligands (A1 – rDIII; B1 – rNadA) or with TBS (negative control, A2 and B2). The interaction was detected using HisProbe-HRP conjugate and visualized with chemiluminescent substrate. Arrow indicates the potential receptors of hBMECs (~15 and ~17 kDa). C shows the alignment of protein marker (lane I, BlueEye prestained protein marker, JenaBioscience), strip transblotted with hBMECs proteins incubated with TBS (lane II, negative control in Western blotting), strip transblotted with hBMECs proteins incubated with recombinant ligand in Western blotting (lane III), strip after acquisition of the chemiluminescent signals from A1 (lane IV), and the nitrocellulose membrane with transblotted proteins of hBMECs, from which 2 mm vertical strip was cut and used in the Western blotting (lane V). Horizontal strip of the nitrocellulose membrane corresponding to the potential receptors of hBMECs (~15 and ~17 kDa) was cut (outlined with horizontal frame). Small piece of horizontal strip was used to affirm the interaction with recombinant ligands. Small piece was either incubated with rDIII (D “+”) or rNadA (E “+”) or TBS (negative controls, D and E “−”) and interaction was detected using HisProbe-HRP conjugate and chemiluminescent substrate. The rest of the strip with potential was used for subsequent identification of putative receptor-binding sites on rDIII and rNadA. Original photos of the blots used to make this figure are presented in the Supplementary Figures S9, S10 and S11.

DIII and NadA were overexpressed in *E. coli*. In short, gene encoding DIII and NadA were cloned into the expression vector pQE-30-mCherry-STOP. *E. coli* were transformed and transformants were selected in the presence of carbenicillin. Overexpressed recombinant proteins were purified with nickel affinity chromatography, ion exchange chromatography and gel filtration. Purity and molecular weights of rDIII and rNadA judged with LDS-PAGE and MALDI-TOF are presented in Supplementary Figure S1. Nucleic acid sequences of the amplified regions of DIII and NadA genes used for ligation are presented in Supplementary Table S1 and S2. Binding of the rDIII and rNadA to the proteins of hBMECs was confirmed first with ELISA. In short, protein extract of hBMECs was coated in microtiter wells. Nonspecific binding sites were blocked and recombinant ligands were added. Unbound proteins were washed and interaction was detected with His-Probe-HRP conjugate and TMB-ELISA substrate. Both rDIII and rNadA showed binding affinity to coated hBMECs proteins (absorbances at 450 nm: 2.2 for rDIII, Fig. 1A and 1.14 for rNadA, Fig. 1B**)**. None of the negative controls showed absorbance more than 0.31 indicating the specific binding of the recombinant ligands to hBMECs proteins. In the Western blotting, both rDIII and rNadA showed binding affinity to low molecular weight proteins (~15 and 17 kDa, respectively) of hBMECs **(**Fig. 2A,B**)**, thus these low molecular weight receptors were used to map binding sites on rDIII and rNadA in further assays. No signal around ~15–17 kDa was observed when recombinant ligands were excluded (negative control).

### Limited tryptic cleavage profiles of rDIII and rNadA

Proteolytic cleavage of the native proteins is limited to the solvent-exposed area, thus several peptides generated with LP do not match with molecular masses of the peptides predicted *in-silico*. Thus, the peptides fingerprints of rDIII and rNadA were generated in *in-solution* LP using trypsin at different time intervals (5 to 60 min). Five peptides of both recombinant ligands obtained from *in-solution* LP coincided with the theoretical masses predicted with *in-silico* trypsin digestion **(**Supplementary Figures S2 and S3**)**. As expected, several peaks did not match with *in-silico* predicted peptide masses, indicating inaccessibility of Arg and Lys residues to trypsin, mainly because of protein folding **(**Supplementary Figures S2 and S3**)**. Note that, trypsin cleaves peptide chains mainly at the carboxyl side of the amino acids Arg or Lys.

### Plausible receptor-binding sites on rDIII and rNadA mapped using NC membrane

Effective elution of the peptides or protein bound to the receptor (immobilized on the NC or PVDF membranes) and their desalting for further MS application were optimized using various stripping buffers and desalting techniques **(**Supplementary Table S3**)**. Conditions were standardized to get maximum recovery of the bound peptides derived from the ligand after digestion with trypsin, with the minimum presence of peptides originated from receptors immobilized on the membrane (data of peptide recovery obtained for each buffer is not shown). In the case of the NC, 25 mM glycine-HCl containing 1% SDS (pH 2.0) stripping buffer followed by acetone precipitation and ZipTip^®^ C4 or C18 showed the highest recovery of the peptides originated from the ligand with the lowest background derived from hBMECs proteins **(**Supplementary Table S3**)**.

**Figure 3:**
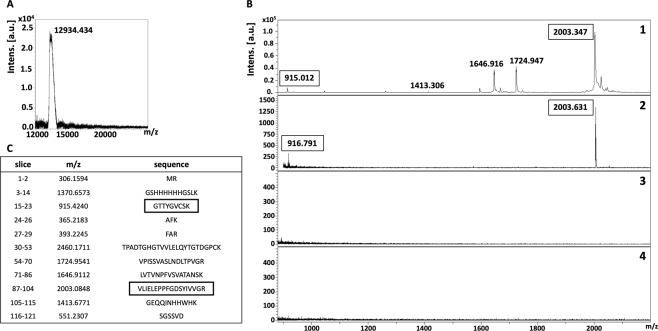
Isolation of putative receptor-binding sites of rDIII using on-membrane limited tryptic digestion (nitrocellulose membrane). (**A**) (1) MALDI spectrum of *in-solution* limited tryptic digestion (20 min digestion) of rDIII, (2) rDIII incubated with ~15 kDa receptor on nitrocellulose membrane was trypsinized, interacting peptides were stripped, acetone precipitated and identified on MALDI-TOF, (3) negative control generated by omitting tryptic digestion, (4) negative control generated by omitting rDIII from the protocol. (**B**) Depicts rDIII recovered after interaction with ~15 kDa receptor from nitrocellulose membrane without trypsinization and detected on MALDI-TOF. C represents list of theoretical peptides of rDIII predicted by *in-silico* tryptic digestion using mMass software. Peptides identified from on-membrane limited proteolysis of ligand-receptor complex (A2), matching with *in-solution* digestion of ligand (A1) and theoretical masses (**C**) are framed. Please note that predicted masses of the peptides are [M + 1 H] +. The observed masses of the peptides are also [M + 1 H] +.

**Figure 4:**
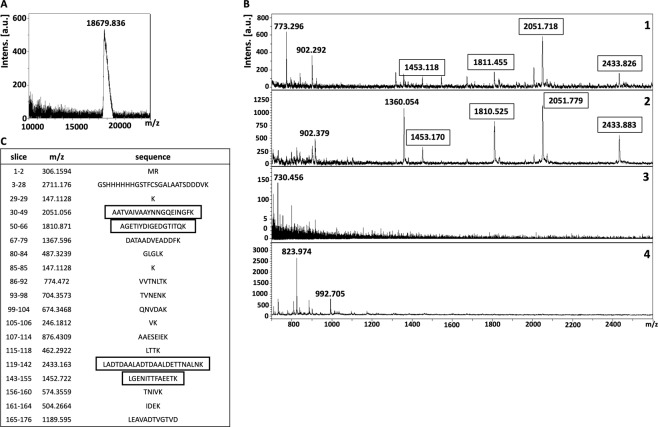
Isolation of putative receptor-binding sites of rNadA using on-membrane limited tryptic digestion (nitrocellulose membrane) (**A**) (1) MALDI spectrum of *in-solution* limited tryptic digestion (20 min digestion) of rNadA, (2) rNadA incubated with ~17 kDa receptor on nitrocellulose membrane was trypsinized, interacting peptides were stripped, acetone precipitated and identified on MALDI-TOF, (3) negative control generated by omitting tryptic digestion, (4) negative control generated by omitting rNadA from the protocol. (**B**) Depicts rNadA recovered after interaction with ~17 kDa receptor from nitrocellulose membrane without trypsinization and identified on MALDI-TOF. C represents list of theoretical peptides of rNadA predicted by *in-silico* tryptic digestion using mMass software. Peptides identified from on-membrane limited proteolysis of ligand-receptor complex (A2), matching with *in-solution* digestion of ligand (A1) and theoretical masses (**C**) are framed. Please note that predicted masses of the peptides are [M + 1 H] +. The observed masses of the peptides are also [M + 1 H] +.

Recovery of the whole recombinant ligand (rDIII and rNadA) bound to the receptor of hBMECs transblotted on the NC membrane was performed as a control. Protein bands were excised (~15 and ~17 kDa, as shown in Fig. 2C) from NC membrane. To confirm that proper bands were excised, a small piece of excised NC strip was incubated either with rDIII (Fig. 2D) or rNadA (Fig. 2E) or TBS (Fig. 2D,E) and interaction was detected using HisProbe-HRP conjugate and chemiluminescent substrate. Once the interaction was confirmed, rest of the NC strip was incubated with the recombinant ligands (rDIII or rNadA), unbound proteins were washed, and bound ligand was stripped, precipitated, desalted and detected with MS. Both rDIII and rNadA were detected on MALDI-TOF **(**Figs. 3A and 4A**)**, which indicates the optimization of the elution and desalting steps.

To map the putative receptor-binding sites on recombinant ligands, trypsin digestion of the ligand-receptor complex (on NC membrane) was subjected for limited trypsin digestion for 20 min, unbound peptides were washed and bound peptides were stripped, precipitated and detected on MALDI-TOF. Two peptides of ~916 Da and ~2003 Da of rDIII were recovered from the NC membrane **(**Fig. 3B**)**. When these molecular masses were correlated with peptides masses obtained from *in-solution* limited tryptic digestion and *in-silico* tryptic cleavage of rDIII (Fig. 3B,C**;** Supplementary Figure S2), a complete match with peptides having sequences GTTYGVCSK (915.424 Da) and VLIELEPPFGDSYIVVGR (2003.084 Da) was found **(**Fig. 3, matched peptides are framed**)**. In case of rNadA, several peptides were recovered, however molecular masses of only 4 peptides (~1453 Da, ~1810 Da, ~2051 Da, and ~2433 Da) were identical with the masses observed in *in-solution* tryptic digest as well as with masses predicted in *in-silico* digestion (LGENITTFAEETK-1452.722 Da, AGETIYDIGEDGTITQK-1810.871 Da, AATVAIVAAYNNGQEINGFK-2051.056 Da and LADTDAALADTDAALDETTNALNK-2433.163 Da) **(**Fig. 4B,C, matched peptides are framed**)**. One peptide of molecular mass ~902 Da was detected in limited tryptic digestion of rNadA complexed with hBMECs receptor on the NC membrane as well as in *in-solution* limited digestion of rNadA, however, it was not predicted in *in-silico* digest **(**Fig. 4B,C**)**. Negative controls generated by omitting either trypsin digestion of ligand-receptor complex or recombinant ligands from the assay showed no peptide coinciding with *in-silico* predicted tryptic cleavage or *in-solution* digest of rDIII or rNadA **(**Figs. 3B and 4B**)**.

### Plausible receptor-binding sites on rDIII and rNadA mapped using PVDF membrane

Instead of using just few protein species of hBMECs excised from the transblotted NC membrane (as presented above), entire protein extract immobilized on the PVDF membrane was used to identify probable hBMECs binding regions of rDIII or rNadA.

**Figure 5:**
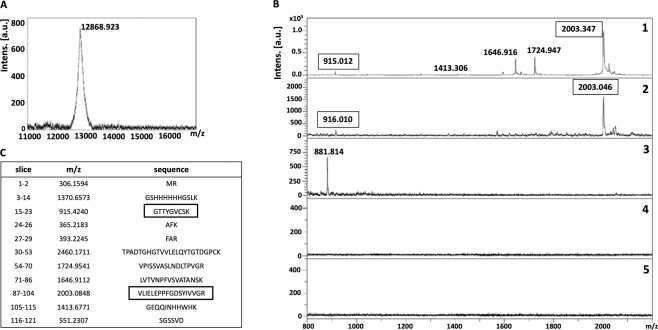
Isolation of putative receptor-binding sites of rDIII using on-membrane limited tryptic digestion (PVDF membrane). (**A**) (1) MALDI spectrum of *in-solution* limited tryptic digestion (20 min digestion) of rDIII, (2) rDIII incubated with proteins of hBMECs immobilized on PVDF membrane was trypsinized, interacting peptides were recovered and identified on MALDI-TOF, (3) negative control generated by omitting tryptic digestion, (4) negative control generated by omitting rDIII from the protocol, (5) negative control generated by omitting proteins of hBMECs from the protocol. (**B**) Depicts rDIII recovered after interaction with proteins of hBMECs immobilized on PVDF membrane without trypsinization and identified on MALDI-TOF. C represents list of theoretical peptides of rDIII predicted by *in-silico* tryptic digestion using mMass software. Peptides identified from on-membrane limited proteolysis of ligand-receptor complex (A2), matching with *in-solution* digestion of ligand (A1) and theoretical masses (**C**) are framed. Please note that predicted masses of the peptides are [M + 1 H] +. The observed masses of the peptides are also [M + 1 H] +.

**Figure 6:**
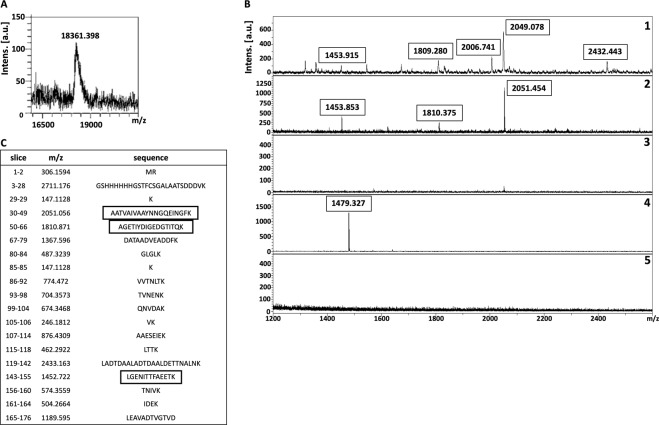
Isolation of putative receptor-binding sites of rNadA using on-membrane limited tryptic digestion (PVDF membrane). (**A**) (1) MALDI spectrum of *in-solution* limited tryptic digestion (20 min digestion) of rNadA, (2) rNadA incubated with proteins of hBMECs immobilized on PVDF membrane was trypsinized, interacting peptides were recovered and identified on MALDI-TOF, (3) negative control generated by omitting tryptic digestion, (4) negative control generated by omitting rNadA from the protocol, (5) negative control generated by omitting proteins of hBMECs from the protocol. (**B**) Depicts rNadA recovered after interaction with proteins of hBMECs immobilized on PVDF membrane without trypsinization and identified on MALDI-TOF. C represents list of theoretical peptides of rNadA predicted by *in-silico* tryptic digestion using mMass software. Peptides identified from on-membrane limited proteolysis of ligand-receptor complex (A2), matching with *in-solution* digestion of ligand (A1) and theoretical masses (**C**) are framed. Please note that predicted masses of the peptides are [M + 1 H] +. The observed masses of the peptides are also [M + 1 H] +.

Recovery of bound recombinant ligands or peptides from the PVDF membrane was also optimized **(**Supplementary Table S3**)**. Incubation of the membrane in formic acid and acetonitrile with sonication, followed by concentration of recovered peptides with vacuum evaporation and desalting by ZipTip^®^ showed the highest efficiency. First, recovery of rDIII and rNadA bound to the proteins of hBMECs immobilized on the PVDF membrane was performed as a control. In short, proteins of hBMECs (whole-cell extract) were immobilized on the PVDF membrane and incubated with recombinant ligands. Unbound proteins were washed and bound ligand was eluted, concentrated, desalted and detected with MALDI-TOF. Detection of rDIII and rNadA **(**Figs. 5A and 6A**)** optimization of the elution buffer and protein recovery steps.

To map the putative receptor-binding sites, rDIII or rNadA bound to the hBMECs (on PVDF membrane) were digested, non-interacting peptides were washed away and bound peptides were recovered. In case of the rDIII, peptides with molecular masses of ~916 Da and ~2003 Da **(**Fig. 5B**)** were identical to those identified earlier using the NC membrane **(**Fig. 3B**)**. Both identified peptides were also matched with predicted molecular masses of the peptide digested *in-silico* **(**Fig. 5C, matched peptides are framed**)**. Likewise, 3 peptides (~1453 Da, ~1810 Da and ~2051 Da, Fig. 6B) of trypsinized rNadA interacting with hBMECs proteins were identical to those recovered using the NC membrane **(**Fig. 4B**)**. The only difference noticed was the absence of ~2433 Da peptide among the peptides recovered from the PVDF membrane, which was found earlier using the NC membrane. Masses of all three peptides identified with MS were matched with molecular masses of the peptide digested *in-silico* **(**Fig. 6C, matched peptides are framed**)**.

Negative controls obtained by omitting either trypsin digestion or recombinant ligands or hBMECs proteins showed almost no peptides (except one each in Figs. 5B and 6B**)**. This indicates no leaching of the peptides derived from hBMECs proteins from PVDF membrane. Two non-specific peptides observed in negative control were neither coinciding with *in-silico* predicted tryptic cleavage nor with peptides derived from the *in-solution* digest of rDIII or rNadA or with the peptides obtained from LP of a ligand-hBMECs protein complex **(**Figs. 5B and 6B**)**.

### Confirmation of putative receptor-binding sites on the ligands using synthetic analogues

**Figure 7:**
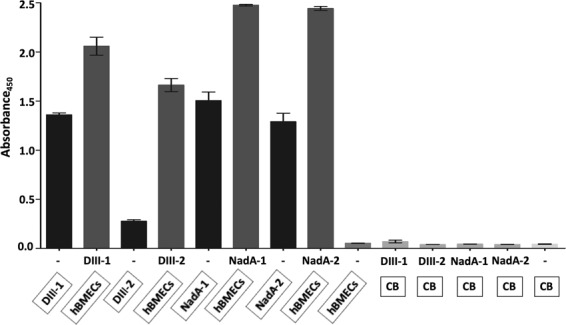
Confirmation of putative receptor-binding sites on the ligands using synthetic analogues by semi-quantitative ELISA. Framed reagents were coated into microtiter wells. Data present means of triplicates with ± S.D. CB – coating buffer; hBMECs – protein extract of human brain microvascular endothelial cells; DIII-1 – GTTYGVCSK-biotin; DIII-2 – VLIELEPPFGDSYIVVGRK-biotin; NadA-1 – AATVAIVAAYNNGQEINGFKAGETIYDIGEDGTITQK-biotin; NadA-2 – LADTDAALADTDAALDETTNALNKLGENITTFAEETK-biotin. Coating of DIII-2 synthetic peptide was very low on the Nunc™ ELISA plate, that is why detection of coated DIII-2 on the plate (input control) with streptavidin-HRP conjugate was weak. Please note that all wells were blocked with blocking buffer after overnight coating. Interaction was detected with streptavidin-HRP conjugate.

**Figure 8:**
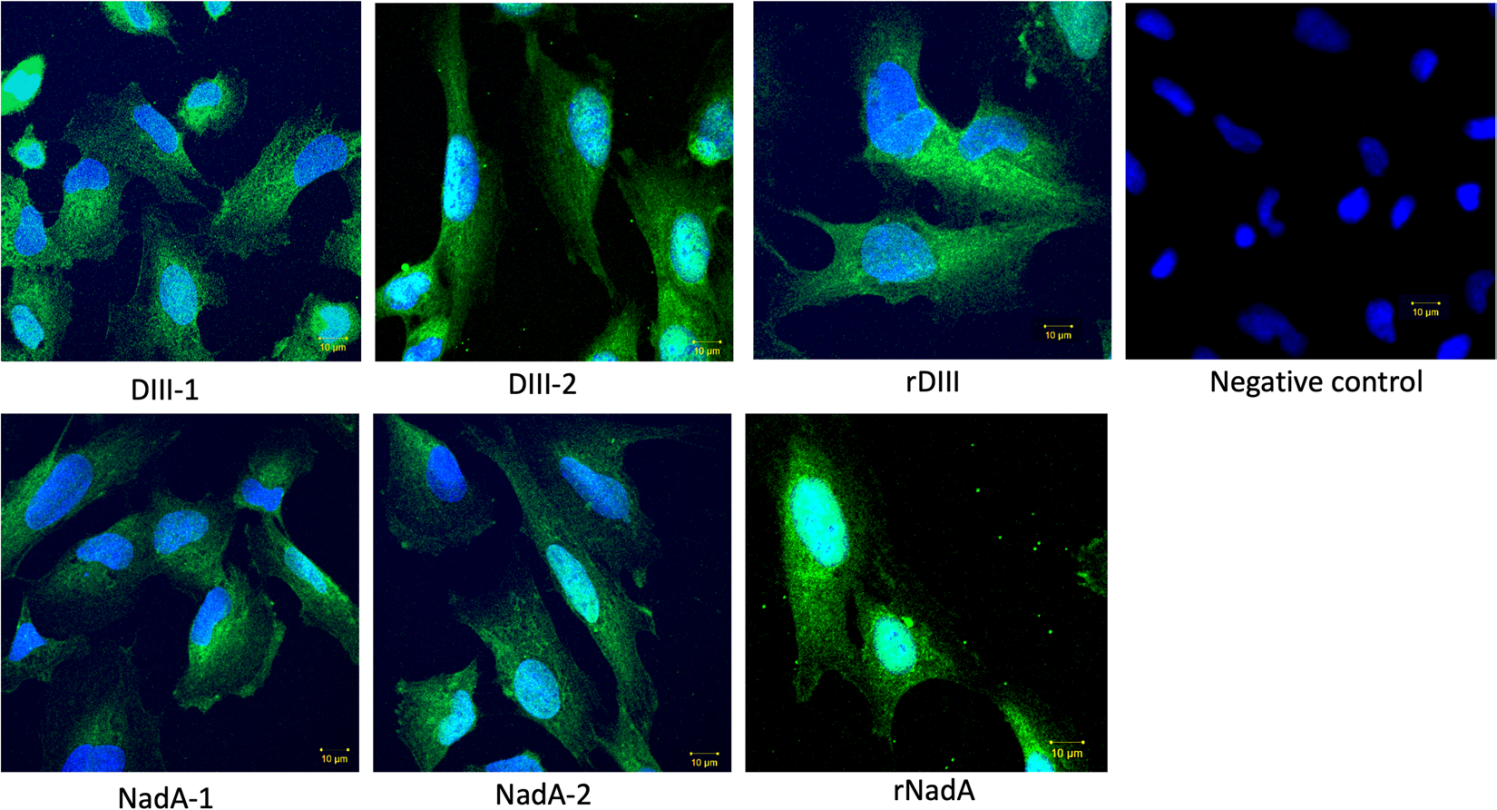
Confirmation of putative receptor-binding sites on the ligands using synthetic analogues by immunocytochemistry. Interaction of synthetic analogues of putative receptor-binding sites with cultured hBMECs. The interaction was detected with streptavidin-FITC conjugate. Nuclei are stained with DAPI. rDIII and rNadA (positive control) – whole recombinant ligands were incubated with hBMECs. DIII-1 – GTTYGVCSK-biotin; DIII-2 – VLIELEPPFGDSYIVVGRK-biotin; NadA-1 – AATVAIVAAYNNGQEINGFKAGETIYDIGEDGTITQK-biotin; NadA-2 – LADTDAALADTDAALDETTNALNKLGENITTFAEETK-biotin. Negative control – synthetic peptides were excluded from the assay.

Synthetic analogues of peptides **(**Supplementary Table S4, Supplementary Figures S4 and S5**)** were used to corroborate binding pockets of the ligands identified above. This validation was necessary to show that: 1. peptides identified in MS are the receptor binders and not from any non-specific origin, and 2. peptides could take part in the formation of putative receptor-binding sites. Both DIII-1 (absorbance 2.059) and DIII-2 (absorbance 1.663) synthetic analogues showed binding affinity to the proteins of hBMECs in ELISA **(**Fig. 7**)**. Similarly, synthetic NadA-1, (which encompasses both AATVAIVAAYNNGQEINGFK-2051.056 Da, AGETIYDIGEDGTITQK-1810.871 Da) and NadA-2 (which encompasses both LGENITTFAEETK-1452.722 Da and LADTDAALADTDAALDETTNALNK-2433.163 Da) retained binding ability to hBMECs proteins **(**Fig. 7**)**. All negative controls showed absorbance < 0.07 which acknowledges the specificity of the interaction **(**Fig. 7**)**. All synthetic analogues also showed binding ability to the endothelial cells in immunocytochemistry, which validates the strategy used to map putative receptor-binding sites on the ligands **(**Fig. 8**)**.

### Location of receptor-binding sites on the ligands

To judge if the receptor-binding sites are surface-exposed, their localization was performed using crystal structures of protein E and NadA in Geneious Pro software. The plausible binding site _299_GTTYGVCSK_307_ (~916 Da peak) of DIII seems to be exposed entirely, whereas the second binding site _371_VLIELEPPFGDSYIVVGR_388_ (~2003 Da peak) is partially exposed on the surface with amino acids – PPFGD and C terminal residues VGR **(**Supplementary Figure S4**)**. In the case of NadA, all lysine residues adjacent to plausible receptor-binding sites are located on the surface, exposed to the solution and accessible to cleavage by trypsin. The binding site, _33_A to _68_K, positioned on the globular domain is also surface exposed and present in the trimeric form. Another amino-acid stretch (_122_L to _158_K), fully exposed to the surface, is on the coiled-coil domain of NadA **(**Supplementary Figure S5**)**. Both binding sites (present on the globular domain and coiled-coiled structure) were cleaved into two peptides because of the presence of internal lysine residues accessible to trypsin. The first of them, _52_K, is located in a turn in the protrusion insertion domain of NadA head, and the second, _145_K, in the trimeric coiled-coil domain **(**Supplementary Figure S5**)**.

## Discussion

Ligand-receptor interactions are pivotal for cell communications and host-pathogen crosstalk. Methods developed to elucidate LRIs include - two-hybrid assays (Y2H or M2H), energy transfer measurements (like fluorescence resonance energy transfer, FRET, or bioluminescence resonance energy transfer, BRET), mutagenesis, phage display, protein array, X-ray crystallography, NMR, and many more^45,46^. Some of them provide high structural resolution with low throughput (e.g. X-ray crystallography and NMR), while others offer low structural resolution but high throughput (e.g. yeast two-hybrid method, protein microarrays, phage display, mass spectrometry, etc.). The best-known method to determine three-dimensional structures - NMR, can be used to study weak LRIs and to molecular details of protein-protein interactions can be witnessed without crystallization. Crystallization remains one of the major hurdles in the X-ray crystallography, while NMR needs a millimolar concentration of proteins^26,47–49^. Both methods are costly (equipment cost) and time-consuming. The mutagenesis, M2H or Y2H assays and FRET are technically complex and require time-consuming genetic manipulations. Two-hybrid and FRET are prone to false-positive results when two proteins of interest are close in distance, but physically do not interact^49,50^. Recent advances in the molecular docking have enabled prediction of interface involved in LRI, however, prediction of the docked complex is possible only when atomic coordinates of both candidates are known^51^. On the other hand, homology modeling (ZDOCK and RDOCK) serve to dock the protein-protein complex based on orthologues/homologues, yet wet-laboratory validation remains inevitable^52,53^. All in all, no technique is fully superior to others. To this background, the present study describes rapid, inexpensive and straightforward pipeline to identify plausible receptor-binding sites on the ligands by using on-membrane limited tryptic digestion of ligand-receptor complex, elution of bound peptides and detection of interacting sites by MS. Our protocol involves immobilization of prey proteins (cell receptors) on a membrane (NC or PVDF) and probing the purified bait proteins (pathogen ligands) with subsequent LP and MS. Some of the advantages of the technique described in this report are: 1. No special matrices are needed to immobilize the prey protein, 2. Although MALDI-TOF is required here, simple MS with linear acquisition is sufficient to detect the peaks derived from LP. 3. The technique requires low protein concentration (1–10 µg), 4. The LP and MS analysis can be performed within a day, 5. The reagents required for the overall method are cheap and available in any MS laboratory, and 6. The method is especially feasible even when the receptors are unknown.

The selection of the DIII and NadA as model proteins was due to the fact that both ligands readily bind receptors (reviewed in^37–39^,) on the host cells. The receptor-binding sites of NadA have been identified experimentally^54^, thus this ligand served to standardize the overall experimental approach and assess the correctness of the developed method. Binding sites of DIII are predicted using bioinformatic tools^55,56^, thus this protein was included in the study as proof of concept.

NadA has been linked to the hypervirulence of *N. meningitidis* that partakes in adhesion of bacteria to the host cells. The crystal structure of NadA has been studied in detail spanning the amino acid residues 28 to 170^57^. The NH2-terminal region (aa 24 to 87, globular structure) was shown to be obligatory for binding the host cells^58^. In another study using NadA mutants, Tavano and colleagues showed that amino acid residues between 24–39 and 88–150 are essential for binding of NadA to the receptor^54^. Our experimental approach also showed that these residues form the plausible receptor-binding site. A peptide with a molecular mass of ~2051 Da encompasses crucial residues (AATVAIVAA) for receptor binding as revealed by Tavano and colleagues. We also found that residues immediate downstream to this region (AGETIYDIGEDGTITQK, ~1810 Da peak) might take part in the interaction. Using mutant proteins, a recent study has shown that residues A33 and Y42 are necessary for the successful interaction of NadA to the mammalian cell surface receptor LOX-1^59^. These residues form the part of NadA peptide 1 in our study. Binding of synthetic analogues **(**Supplementary Table S4, Supplementary Figure S5**)** to the endothelial cells was confirmed in both ELISA and immunocytochemistry **(**Figs. 7 and 8**)**, which validates the overall methodological approach employed to map the binding sites in LRIs.

Receptor binding sites on DIII are predicted in earlier studies^55,56,60^. In particular, residues around K307 and E390 are described to form receptor-binding regions^55,56,60,61^. It was shown that residues near K307 and E390, located on the distal lateral face of domain III, could be the key determinants for glycosaminoglycans binding affinity^62^. Both peptides ~916 Da and ~2003 Da found in our study lie around K307 and E390 **(**Supplementary Figure S4**)**. These residues are located in close proximity to each other at the tip of two surface-exposed loops in domain III^63,64^. It was shown that changes in these residues affect virulence and receptor binding^64,65^, underlying their importance in the formation of the interface in LRI. In ELISA and immunocytochemistry, synthetic analogues of both ~916 Da and ~2003 Da peptides showed binding affinity to the hBMECs **(**Figs. 7 and 8**)**. These data again confirm the appropriateness of the method employed to map the receptor-binding site of DIII.

In this study, two different experimental approaches were tested for the identification of plausible receptor-binding sites of the pathogen ligands. In the first strategy, prey proteins were fractionated with LDS-PAGE, transblotted on the NC membrane and allowed to form a complex with bait protein. Separation of pray proteins by SDS-PAGE usually results in denaturation of proteins by SDS^66^ that could lead to the loss of secondary and tertiary structures compromising protein binding ability. Thus, mild denaturation conditions were used here by replacing SDS with lithium dodecyl sulfate (LDS) in sample buffer and omitting heat denaturation. However, it cannot be ruled out that some prey proteins might get denaturated. Thus, in the second strategy immobilization of prey protein was achieved by direct spotting of the cell lysate (extracted in native conditions) on the PVDF membrane.

Different stripping buffers were tested in the study to achieve maximum recovery of the peptides derived from bait proteins (receptor-binding sites) with the least presence of peptides originated from the prey protein immobilized on the membrane. We found that 25 mM glycine-HCl with 1% SDS (pH 2.0) for the NC membrane, and low pH buffer made up of formic acid and acetonitrile for the PVDF membrane were the best suitable recovery buffers as very few non-specific peptide species were released from the membranes (Supplementary Table S3; Figs. 3A to 6A**)**. Stripping involves the disruption of the interaction between membrane-bound protein(s) and bait protein, which can be achieved by using an appropriate combination of detergents, heat, time, and pH. Generally, the low pH glycine-HCl does not strip the transblotted proteins from the membrane, however, the recovery of the bait protein/antibody is usually partial^67^. The use of 10% SDS usually leads to complete recovery of bait protein when combined with heating at 50 °C to 70 °C. However, it also accompanies with the stripping of transblotted proteins^67^. Moreover, we observed that 10% SDS in the stripping buffer caused aggregates formation during the acetone precipitation, which was difficult to remove for MALDI-TOF analysis (data not shown). Thus, we reduced the concentration of SDS to 1% in low pH glycine-HCl buffer, which led to negligible leaching of transblotted hBMECs proteins or its peptides from the NC membrane. For example, in some replicates very few peptides of molecular mass < 1000 Da were observed in negative control in which the bait ligand was omitted **(**Fig. 4B**)**. In case of the PVDF membrane, the treatment of formic acid and acetonitrile with subsequent sonication was proved the optimum strategy to recover the peptides of the bait ligands bound to the proteins of hBMECs. While in the negative controls, wherein bait proteins were omitted from the experiment, only one non-specific peptide (~1479 Da) was found to be leached in some replicates from the hBMECs immobilized on the PVDF membrane **(**Fig. 6B**)**. Formic acid helps to break down the protein-protein interaction, while acetonitrile promotes solubility of the peptides. Although the majority of peptides should be stripped in formic acid and acetonitrile solution, additional sonication ensured maximum recovery.

Several protein-based blocking agents (albumin, ovalbumin, gelatin, skimmed milk, etc.) are routinely used in far-western blotting techniques or protein-protein interaction assays. However, the use of proteinous agents was not possible, as peptides derived from those agents could be the major source of non-specific contaminants in MS. Therefore, polymer-based blocking buffer (polyvinylpyrrolidone, PVP) was used to block the NC membrane. Polymers such as polyethylene glycol (PEG)^68^, polyvinyl alcohol^69^, polyvinyl alcohol-glutaraldehyde^70^, and PVP^71,72^ have been used before without cross-reactivity or interference with specific binding. PVP and PEG do not mask the bound proteins^71,73^, which enhances the sensitivity and specificity of the blotting assays^63,64^. In contrast to the NC membrane, the PVDF membrane does not require blocking because of its highly hydrophobic nature, which does not allow non-specific binding of baited proteins.

Limited proteolysis coupled with MALDI-TOF has been employed previously for mapping of epitopes and DNA-binding proteins^74,75^. In the present study, we performed time-dependent *in-solution* LP of bait proteins to get peptide spectra **(**Supplementary Figures S2 and S3**)**, which were used later to compare with the peaks obtained from on-membrane LP of ligand-receptor complex. Please note that *in-silico* digestion of rDIII and rNadA was also performed in the present study **(**Figs. 3C to 6C**)** to generate a reference peak list of the peptides, from virtually digested recombinant ligands. Finally, the peak spectra obtained from LP experiments were compared to the peak list derived from *in-silico* digestion **(**Figs. 3 to 6**)**. This comparison is necessary because *in-silico* digestion is based on the linearized protein sequence, whereas *in-solution* digestion generates a peptide spectrum based on the availability of cleavage sites. The availability of cleavage sites is influenced by intrinsic factors like protein folding and structure, stability and post-translational modifications^76^.

It is important to note that, the experimental approach of LP of the ligand-receptor complex presented here might not reveal all binding sites on the ligand especially when the binding site contains multiple arginine or lysine residues. This leads to fragmentation of the receptor-binding site into smaller fragments (<800 Da) after digestion with trypsin, which are difficult to detect with MALDI-TOF with sufficient confidence. We noticed that in case of rNadA, we were not able to detect the peak that corresponds to the receptor-binding site predicted previously using NadA mutants^54^ (this site encompasses aa 90–120, and is marked in Supplementary Figure S5). It might be because of the presence of multiple surface-exposed lysine residues in this region. This pitfall can be overcome by using different proteolytic enzyme like chymotrypsin or ArgC or AspN.

The overall data suggest that the experimental approach described in the present study can be used as a potential screening method to assess plausible receptor-binding sites with minimum time, cost and labour, and could be a simpler complementary method to the existing techniques used to study ligand-receptor interface.

## Methods

### Extraction of proteins of hBMECs

hBMECs (D3 cell line) were cultured as described by Jimenez-Munguia *et al*.^77^. Details are presented Supplementary Method S1.

### Preparation of recombinant ligands

Gene fragments encoding DIII of the envelope protein of WNV (strain goshawk, Hungary) and globular domain with the first coiled-coil structure of NadA of *N. meningitidis* (isolate M1/03, Czech Republic) were PCR amplified (primers in Supplementary Table S5**)**. PCR products were cloned into a pQE-30-mCherry-STOP expression vector. Details of the vector, ligation and transformation of *E. coli* and protein overexpression are presented in Supplementary Figure S6 and Supplementary Method S2. Details of the protein purification are presented in Supplementary Method S3, whereas, the methods used to check the purity of recombinant proteins (LDS-PAGE and MALDI-TOF MS) are described in Supplementary Method S4.

### Interaction of recombinant ligands and proteins of hBMECs

The binding ability of the recombinant ligands was judged by ELISA and Western blotting. Details of both methods are described in Supplementary Methods S5 and S6.

### Isolation of putative receptor-binding sites of recombinant ligands using on-membrane limited tryptic digestion (nitrocellulose membrane)

A protein band of hBMECs that interacts with rDIII and rNadA was excised from the NC membrane as described in Fig. 2C. A small piece of the cut membrane was used to confirm the interaction between the receptor of hBMECs and recombinant ligands (rDIII or rNadA) by Western blotting as described in Supplementary Method S6. Once the interaction was confirmed, the excised NC band was blocked in 1% PVP-40 in TBS and then incubated with rDIII or rNadA (5 µg of purified rDIII or rNadA for 3 h at room temperature). After 3 washings with TBST-20 each for 5 min, on-membrane trypsinization was performed by incubating the excised NC band with 1 µg of Trypsin Gold (Promega, USA) diluted in 300 µl of pre-warmed (37 °C) ammonium bicarbonate (50 mM, pH 7.8, Sigma) for 20 min at 37 °C. The NC strip was washed 3 times with TBST-20 to eliminate non-interacting peptides. Interacting peptides were stripped from the NC membrane by immersing it into 500 µl of stripping buffer (25 mM glycine-HCl + 1% SDS, pH 2.0) for 45 min at room temperature with gentle shaking. Stripped peptides were precipitated with 2 ml of acetone for 60 min at room temperature with subsequent centrifugation at 15000 × *g* for 15 min. The pellet was air-dried and resuspended in 5 µl of TA50 (50:50 [v/v] acetonitrile: 0.1% trifluoroacetic acid TFA, Sigma). After complete resuspension, 25 µl of 0.1% TFA was added. Peptides were desalted and concentrated with ZipTip^®^ C18 (Millipore, USA) following the manufacturer’s instructions. Peptides were eluted in α-Cyano-4-hydroxycinnamic acid (HCCA; Bruker-Daltonics) matrix dissolved in TA30 solvent (30:70 [v/v] acetonitrile: 0.1% TFA). Two microliters of the eluate were spotted on AnchorChip (Bruker-Daltonics) and air-dried. The spectra were analyzed in MALDI-TOF Microflex^TM^-LRF mass-spectrometer (Bruker-Daltonics). Peptide calibration standard II (Bruker-Daltonics) was used as a calibrant. Spectra were acquired in reflectron-positive mode at a laser frequency of 35 Hz (100 to 200 shots). For negative controls, either the on-membrane tryptic digestion step or recombinant ligands were excluded from the protocol.

To show that the recombinant ligand was interacting with the receptor of hBMECs (input control), rDIII or rNadA were incubated with the excised NC band as described above and washed 3 times with TBST-20. The bound recombinant ligand was stripped from the NC membrane using stripping buffer, acetone precipitated, and desalted using ZipTip^®^C4. Proteins were eluted in sDHB (2,5-dihydroxybenzoic acid and 2-hydroxy-5-methoxybenzoic acid, Bruker-Daltonics) matrix dissolved up to saturation in TA50. The eluate was spotted on ground steel target plate (Bruker-Daltonics), allowed to air dry and ligands were detected in linear-positive mode on MALDI-TOF.

### Isolation of putative receptor-binding sites of recombinant ligands using on-membrane limited tryptic digestion (PVDF membrane)

Two micrograms of hBMECs protein extract were spotted on activated Immobilon-FL PVDF membrane (Millipore) and air-dried overnight. The membrane was submerged in TBS (pH 7.2) contacting 5 µg of recombinant ligand for 3 h at room temperature with shaking. The membrane was washed with TBS (6 times, each for 1 min) and dried overnight prior to tryptic digestion. Limited trypsin digestion of the bound recombinant ligand was performed with 1 µg of Trypsin Gold dissolved in pre-warmed ammonium bicarbonate for 20 min. Non-interacting fragments of the digested recombinant ligand were removed by washing with ammonium bicarbonate (6 times). To elute interacting peptides from the PVDF, the membrane was vortexed for 2 min in 10 µl of ~98% formic acid (Sigma), followed by addition of 50 µl of ≥99.9% acetonitrile (Sigma), and sonication in sonicator-bath for 15 min. The supernatant was retrieved, vacuum dried at 60 °C and pellet was resuspended in 5 µl of TA50. After complete resuspension, 25 µl of 0.1% TFA was added. Peptides were desalted with ZipTip^®^C18, eluted in HCCA matrix dissolved in TA30, and peptides were detected on MADI-TOF in positive mode using AnchorChip as described above. For negative controls, either tryptic digestion step or recombinant ligands or hBMECs protein lysate were omitted from the protocol.

To show that recombinant ligands were interacting with hBMECs protein extract (input control), rDIII or rNadA were incubated with hBMECs spotted on PVDF membrane as described above and washed 6 times with TBS. The bound recombinant ligand was eluted using formic acid and acetonitrile, desalted with ZipTip^®^C4 and eluted in sDHB matrix dissolved up to saturation in TA50 as mentioned above. The eluate was spotted on ground steel target plate, allowed to air dry and ligand was detected in linear-positive mode on MALDI-TOF.

### Limited tryptic digestion of recombinant ligands (*in-solution*)

Ten micrograms of rDIII or rNadA were diluted in 50 µl of pre-warmed ammonium bicarbonate, 1 microgram of Trypsin Gold was added and incubated at 37 °C for different time intervals (5 to 60 min) as shown in Supplementary Figures S2 and S3. Digestion was stopped by placing the tubes at −20 °C until MS. Digested proteins were diluted (1:2 v/v) with 0.1% TFA. ZipTip^®^C18 was used to desalt digested peptides and peptides were eluted in HCCA dissolved in TA30. Two microliters of the eluate were spotted on AnchorChip and air-dried. The spectra were analyzed in MALDI-TOF either in linear-positive or reflectron-positive mode. Peptide calibration standard II was used as a calibrant.

### Mapping of the putative receptor-binding sites of recombinant ligands

First, *in-silico* prediction of the tryptic digestion of rDIII and rNadA was performed in mMass software (http://www.mmass.org/) using following conditions: miscleavage – 0, monoisotopic mass, maximum charge 1. Subsequently, peptide spectra obtained from the limited tryptic digestion of rDIII or rNadA-hBMECs receptor complex (from the NC and PVDF membranes) were aligned with spectra obtained from *in-solution* digestion of the recombinant ligands using flexAnalysis (V 3.4, Bruker-Daltonics). Aligned peptide masses were correlated with the list of peptides obtained from *in-silico* tryptic digestion of rDIII or rNadA.

### Confirmation of interaction between putative receptor-binding sites and proteins of hBMECs

Putative receptor-binding sites identified above were synthesized commercially (Caslo, Denmark). Peptides were biotinylated in C-terminal lysine. Peptides were dissolved according to the manufacturer’s instructions **(**Supplementary Table S4**)**. ELISA assay was employed to corroborate interaction between peptides and proteins of hBMECs (details are in Supplementary Method S7**)**.

### Confirmation of interaction between putative receptor-binding sites and cultured hBMECs

hBMECs were cultured on the coverslips coated with collagen type I (Sigma) in 12 well culture plate in EBM-2 medium (Lonza) as was described earlier^77^ until 70% confluency. Cells were washed 2 times with PBS (pH 7.4) and fixed with 4% paraformaldehyde for 15 min at 37 °C. Cells were washed 3 times with PBS and incubated with 2 μg of a synthetic peptide in PBS for 1 h at room temperature with gentle shaking. After 4 washings, cells were incubated with Streptavidin-FITC conjugate (diluted in PBS 1:500, Rockland Immunochemicals, USA) for overnight in dark at 4 °C with gentle shaking. After 3 washings with PBST-20 (5 min each) and 1 with PBS, coverslips were taken out of the wells, dipped in ethanol for 2–3 sec and mounted using Fluoroshield containing DAPI (Sigma). Scanning was performed on LSM-710 microscope (Zeiss, Germany) using 359–461 nm filter for DAPI and 495–519 nm filter for FITC. The assay was performed in biological triplicates. As a positive control, rDIII and rNadA (20 µg diluted in 1 ml of PBS) were used in the assay and their binding on hBMECs was detected with anti-6x His antibody^®^ conjugated with FITC (diluted in PBS with 1% BSA, 1:500, Abcam, UK). In the case of negative control, synthetic peptides were excluded from the experiment.

### Localization of putative receptor-binding sites on crystal structures of DIII and NadA

Crystal structures of protein E (2HG0) and NadA (6EUP) were retrieved from PDB (www.rcsb.org). Amino acid sequences of the receptor-binding sites identified in this work were localized in the structures using Geneious Pro 9.0 (www.geneious.com). To show the location of binding sites, amino acid sequences of protein E (NC_009942) and NadA (WP_01098101) were retrieved of NCBI and aligned with sequences used to design rDIII and rNadA using ClustalW in Geneious Pro software.

## Supplementary information

Supplementary information.

## Data Availability

Data generated in this study are available from the corresponding author on reasonable request.
