# Supplementary material for: A simple and rapid pipeline for identification of receptor-binding sites on the surface proteins of pathogens
Source: Sci Rep. 2020 Jan 24;10:1163. doi: 10.1038/s41598-020-58305-y (PMC6981161; doi:10.1038/s41598-020-58305-y)
Supplement: Supplementary file 1 — Supplementary information. [file 41598_2020_58305_MOESM1_ESM.pdf]

A simple and rapid pipeline for identification of receptor-binding sites on the surface proteins of pathogens

Patrícia Mertinková[1], Amod Kulkarni[1,2], Evelína Káňová[1], Katarína Bhide[1], Zuzana Tkáčová[1], Mangesh Bhide[1,2*]

[1]Laboratory of Biomedical Microbiology and Immunology, The University of Veterinary Medicine and Pharmacy, Komenskeho 73, 04001, Kosice, Slovakia,

[2]Institute of Neuroimmunology of Slovak Academy of Sciences, 84510, Bratislava, Slovakia

SUPPLEMENTARY FIGURES

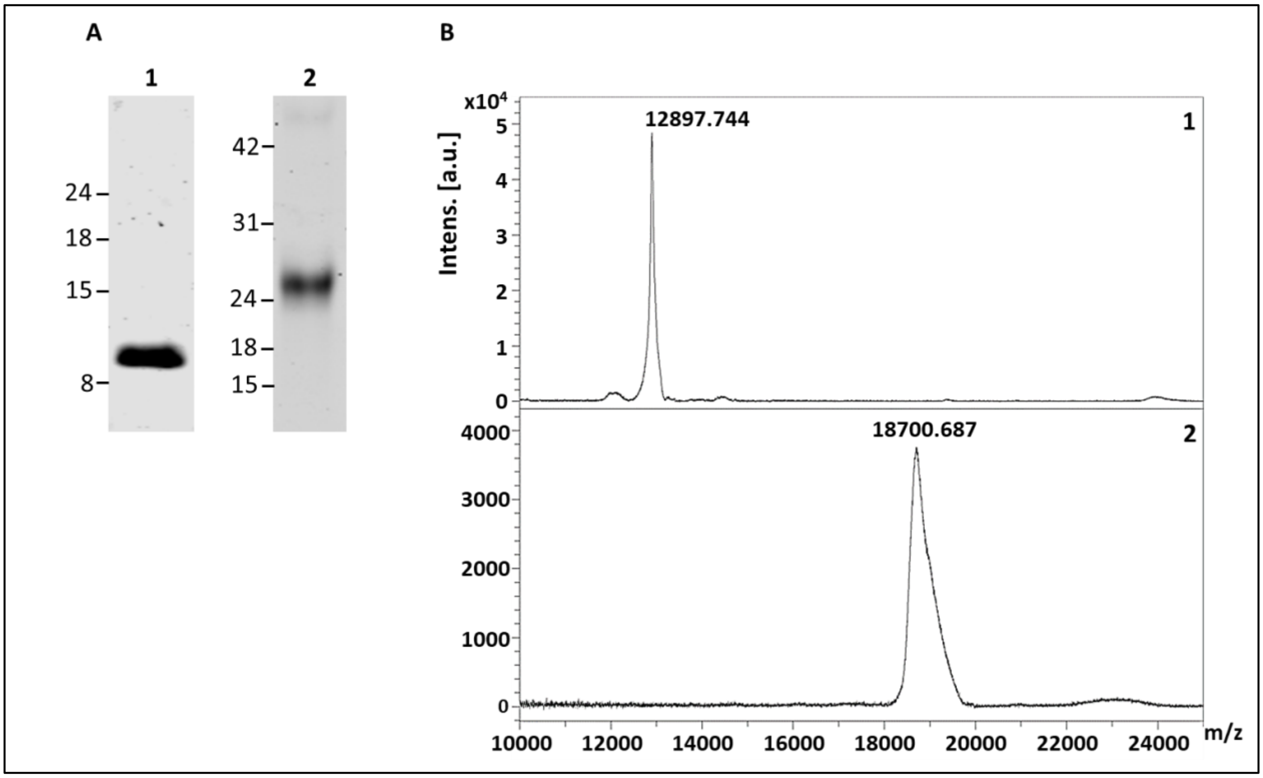

Supplementary Figure S1. Production of recombinant form of DIII and NadA. *A shows purified recombinant ligands separated with LDS-PAGE (1 – rDIII; 2 – rNadA). B represents confirmation of molecular masses of rDIII (1) and rNadA (2) by MALDI-TOF mass spectrometry. Please note that the predicted molecular mass of rNadA is 18.7 kDa which was also confirmed by MALDI-TOF mass spectrometry (B2), however it shows slower migration on LDS-PAGE (nearly ~25 kDa).*

A

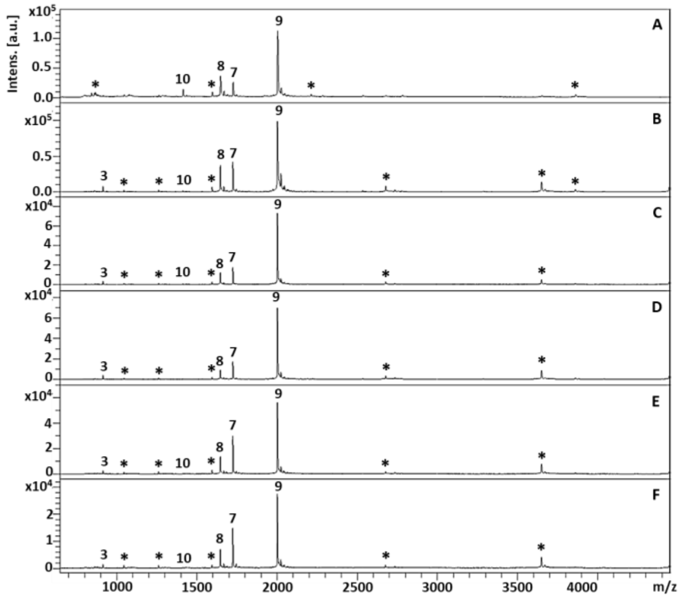

B

no.	slice	m/z	sequence
1	1-2	306.1594	MR
2	3-14	1370.6573	GSHHHHHHGSLK
3	15-23	915.4240	GTTYGVCSK
4	24-26	365.2183	AFK
5	27-29	393.2245	FAR
6	30-53	2460.1711	TPADTGHGTVVLELQYTGTDGPCK
7	54-70	1724.9541	VPISSVASLNDLTPVGR
8	71-86	1646.9112	LVTVNPFVSVATANSK
9	87-104	2003.0848	VLIELEPPFGDSYIVVGR
10	105-115	1413.6771	GEQQINHHWHK
11	116-121	551.2307	SGSSVD

Supplementary Figure S2. Limited tryptic digestion of rDIII at different time intervals (*in solution* tryptic digestion). *A shows MALDI spectra obtained after 10 min (A); 20 min (B), 30 min (C), 40 min (D), 50 min (E), and 60 min (F) of in solution limited tryptic digestion of rDIII at 37°C. B depicts list of theoretical peptides predicted after in-silico tryptic digestion of rDIII using mMass software. Numbered peaks corresponding to theoretically predicted peptides. "*" represents peaks that do not match with any peptide mass predicted in-silico. These peaks represent peptides that are not completely digested due to inaccessibility of the arginine or lysine (mainly because of protein folding). Please note that predicted masses of the peptides are [M+1H]+. The observed masses of the peptides are also [M+1H]+.*

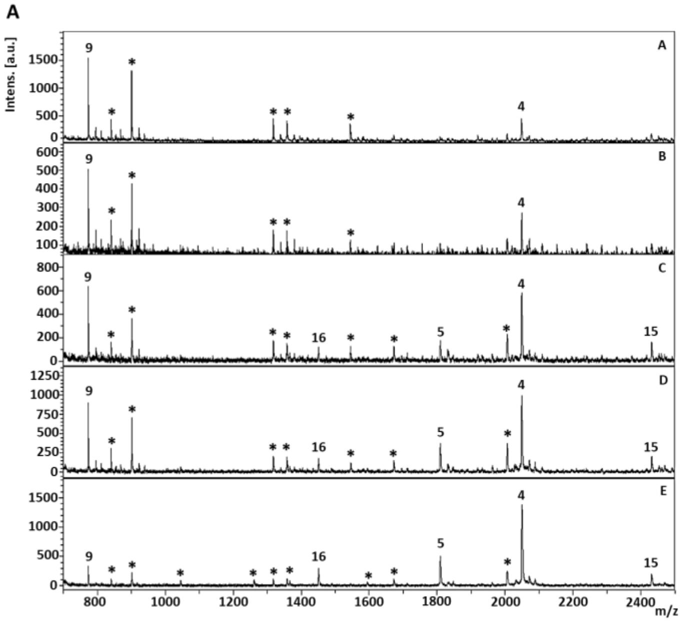

B

no.	slice	m/z	sequence
1	1-2	306.1594	MR
2	3-28	2711.176	GSHHHHHHGSTFCSGALAATSDDDVK
3	29-29	147.1128	K
4	30-49	2051.056	AATVAIVAAYNNGQEINGFK
5	50-66	1810.871	AGETIYDIGEDGTITQK
6	67-79	1367.596	DATAADVEADDFK
7	80-84	487.3239	GLGLK
8	85-85	147.1128	K
9	86-92	774.472	VVTNLTK
10	93-98	704.3573	TVNENK
11	99-104	674.3468	QNVDAK
12	105-106	246.1812	VK
13	107-114	876.4309	AAESEIEK
14	115-118	462.2922	LTTK
15	119-142	2433.163	LADTDAALADTDAALDETTNALNK
16	143-155	1452.722	LGENITTFAEETK
17	156-160	574.3559	TNIVK
18	161-164	504.2664	IDEK
19	165-176	1189.595	LEAVADTVGTVD

Supplementary Figure S3. Limited tryptic digestion of rNadA at different time intervals (*in solution* tryptic digestion). *A shows MALDI spectra obtained after 5 min (A); 10 min (B), 20 min (C), 30 min (D), and 60 min (E) of in solution limited tryptic digestion of rNadA at 37°C. B depicts list of theoretical peptides predicted after in-silico tryptic digestion of rNadA using mMass software. Numbered peaks corresponding to theoretically predicted peptides. "*" represents peaks that do not match with any peptide mass predicted in-silico. These peaks represent peptides that are not completely digested due to inaccessibility of the arginine or lysine (mainly because of protein folding). Please note that predicted masses of the peptides are [M+1H]+. The observed masses of the peptides are also [M+1H]+.*

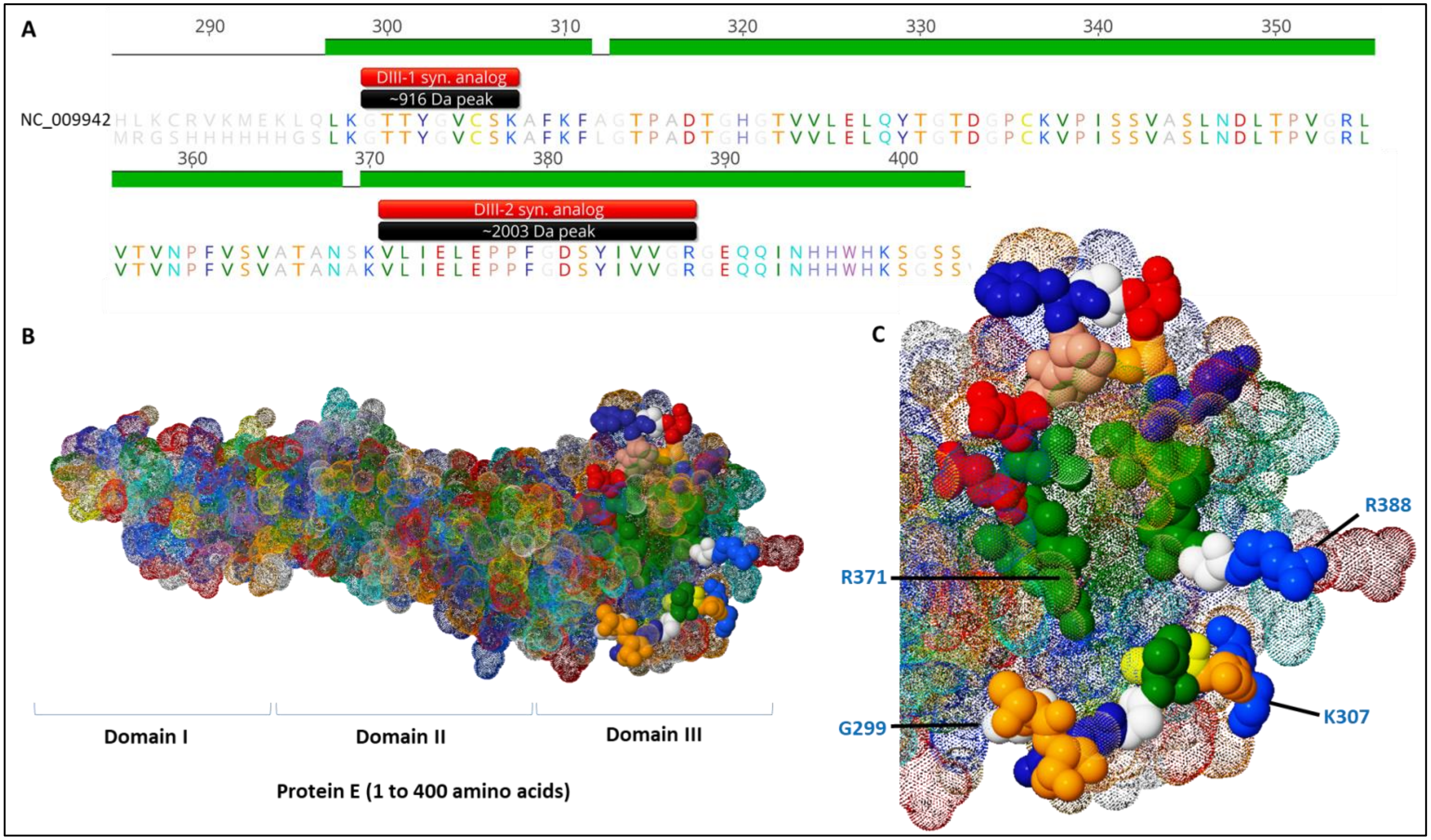

Supplementary Figure S4. Location of the putative receptor-binding sites on DIII. *A shows amino acid sequence alignment of the DIII used in the study (lower sequence in the alignment) with the sequence deposited under NC_009942 in the Genbank. Green color indicate consensus. Black annotations - plausible receptor-binding sites on DIII detected in this study by LP and mass spectrometry. It also shows m/z of the peak observed in MALDI-TOF. Red annotations - amino acid sequence and designation of synthetic analogue used to corroborate interaction between binding site and the hBMECs using ELISA and ICC. B presents crystal structure of protein E (PDB accession number 2HG0), in which plausible receptor-binding sites are highlighted. C – zoom in of the DIII, in which amino acids positions of receptor-binding sites identified in the study are marked.*

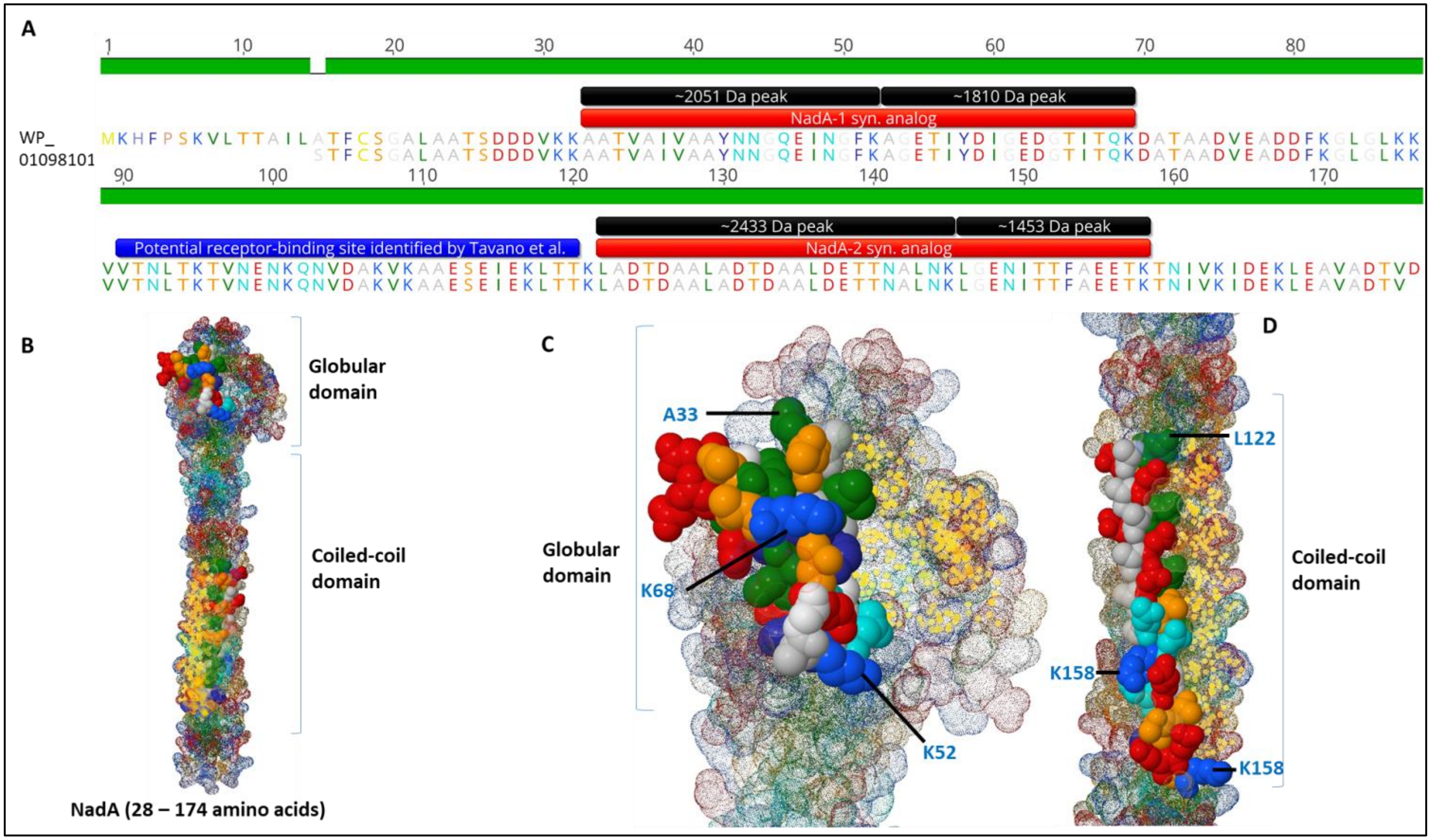

Supplementary Figure S5. Location of the putative receptor-binding sites on NadA. *A shows amino acid sequence alignment of the NadA used in the study (lower sequence in the alignment) with the sequence deposited under WP_01098101 in the Genbank. Green color indicate consensus. Black annotations - plausible receptor-binding sites on NadA detected in this study by LP and mass spectrometry. It also shows m/z of the peak observed in MALDI-TOF. Red annotations - amino acid sequence and designation of synthetic analogues used to corroborate interaction between binding sites and hBMECs using ELISA and ICC. Please note the potential binding site proposed by Tavano et al. (doi: 10.1128/JB.00430-10) enriched in lysine residues (annotated in blue color). B presents crystal structure of NadA (PDB accession number 6EUP), in which plausible receptor-binding sites are highlighted. Zoom in of the globular domain (C) and coiled-coil domain (D), in which amino acids positions of receptor-binding sites identified in the study are*

marked. Please note that NadA is present in trimeric form. Amino acids that forms putative receptor-binding site in other monomer are highlighted with yellow dots.

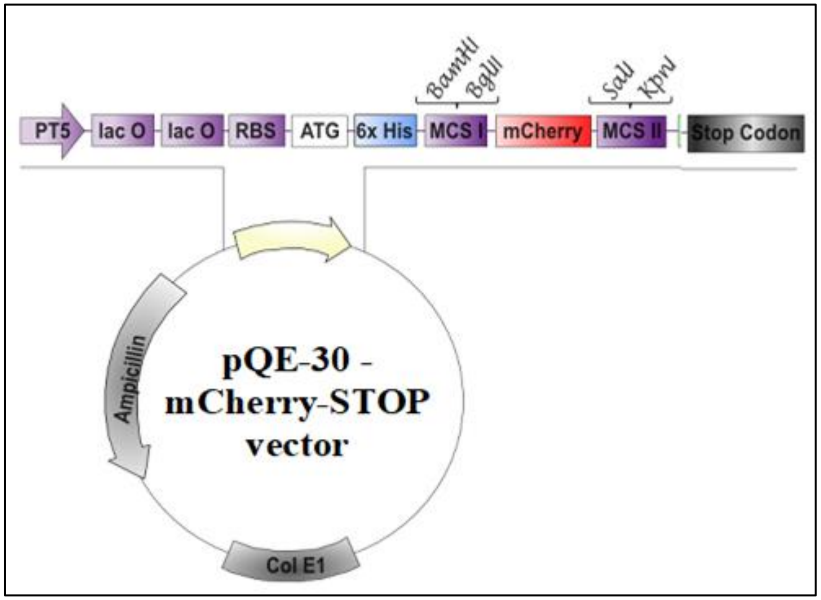

Supplementary Figure S6. pQE-30-mCherry-STOP plasmid (4880 bp). *Col E1 – origin of replication, ampicillin – resistance gene, PT5 – T5 promoter, lac O – lac operator, RBS – ribosome binding site, ATG – start codon, 6x his-tag sequence, MCSI/MCSII – multiple cloning sites, mCherry – red fluorescent protein that serves as stuffer, stop codon.*

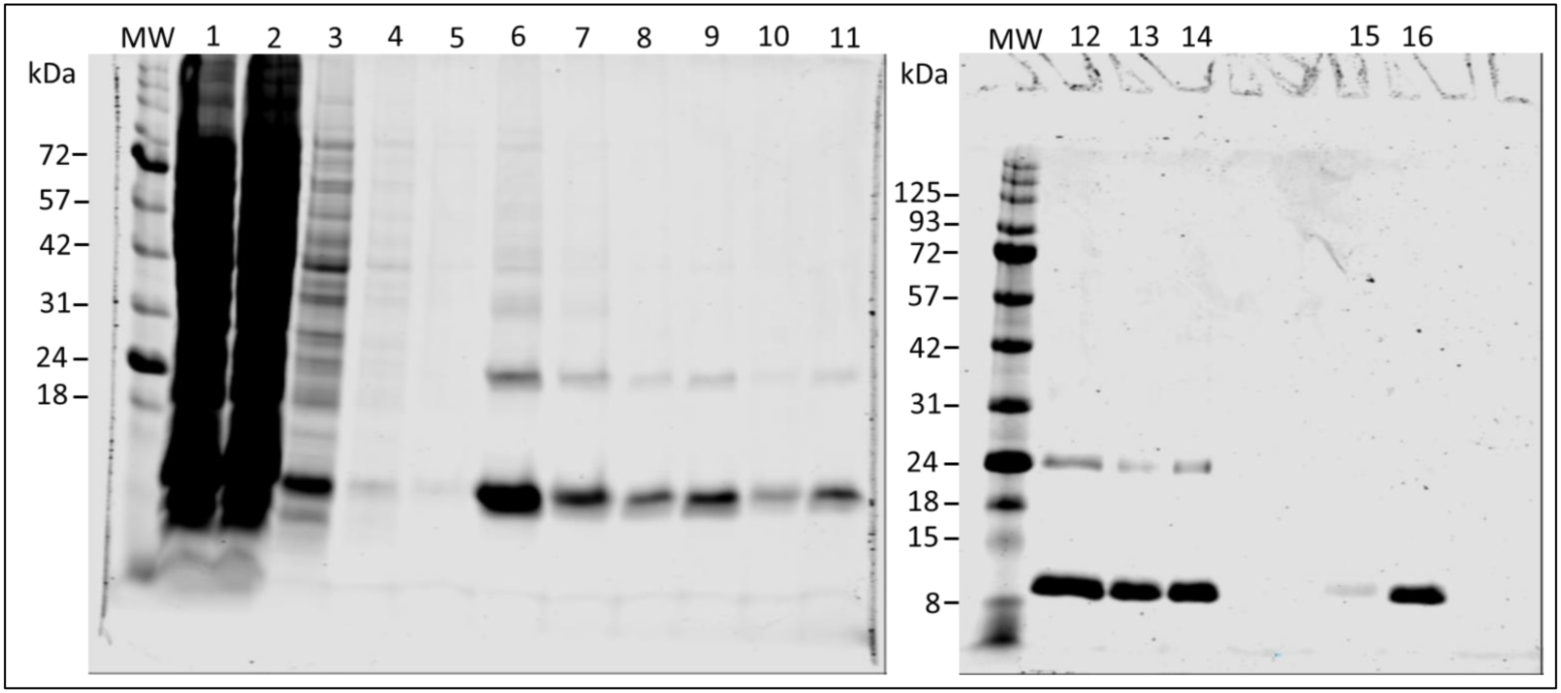

Supplementary Figure S7. Original figures of the gels used to make the Supplementary Figure S1. *Purified recombinant DIII of West Nile virus envelope protein separated on LDS-PAGE. MW – molecular weight marker; lanes 1 – whole-cell lysate of E. coli overexpressing rDIII; lane 2 – flow-through obtained during nickel affinity chromatography; lane 3 – 1st washing of the nickel affinity beads after protein binding; lanes 4 and 5 – subsequent washing of the beads; lanes 6 - 11 – step wise elution performed with increasing concentrations of imidazole; lanes 12 - 13 – elute from lanes 6 to 11 after concentration; lanes 15 - 16 – rDIII eluted from MWCO gel filtration.*

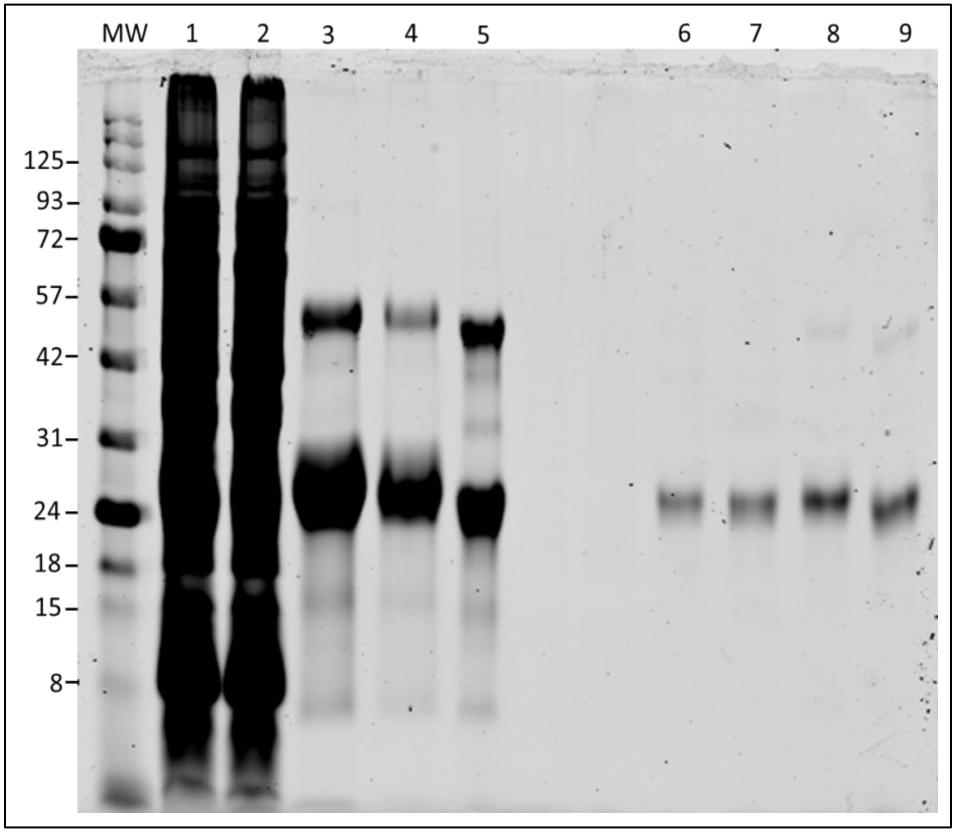

Supplementary Figure S8. Original figure of the gel used to make the Supplementary Figure S1. *Purified recombinant NadA separated on LDS-PAGE. MW – molecular weight marker; lane 1 – whole-cell lysate of E. coli overexpressing rNadA; lane 2 – flow-through; lane 3 – 1st elution after nickel affinity chromatography; lane 4 – 2nd elution after nickel affinity chromatography; lane 5 – rNadA after gel filtration; lanes 6 - 9 – rNadA after anion exchange.*

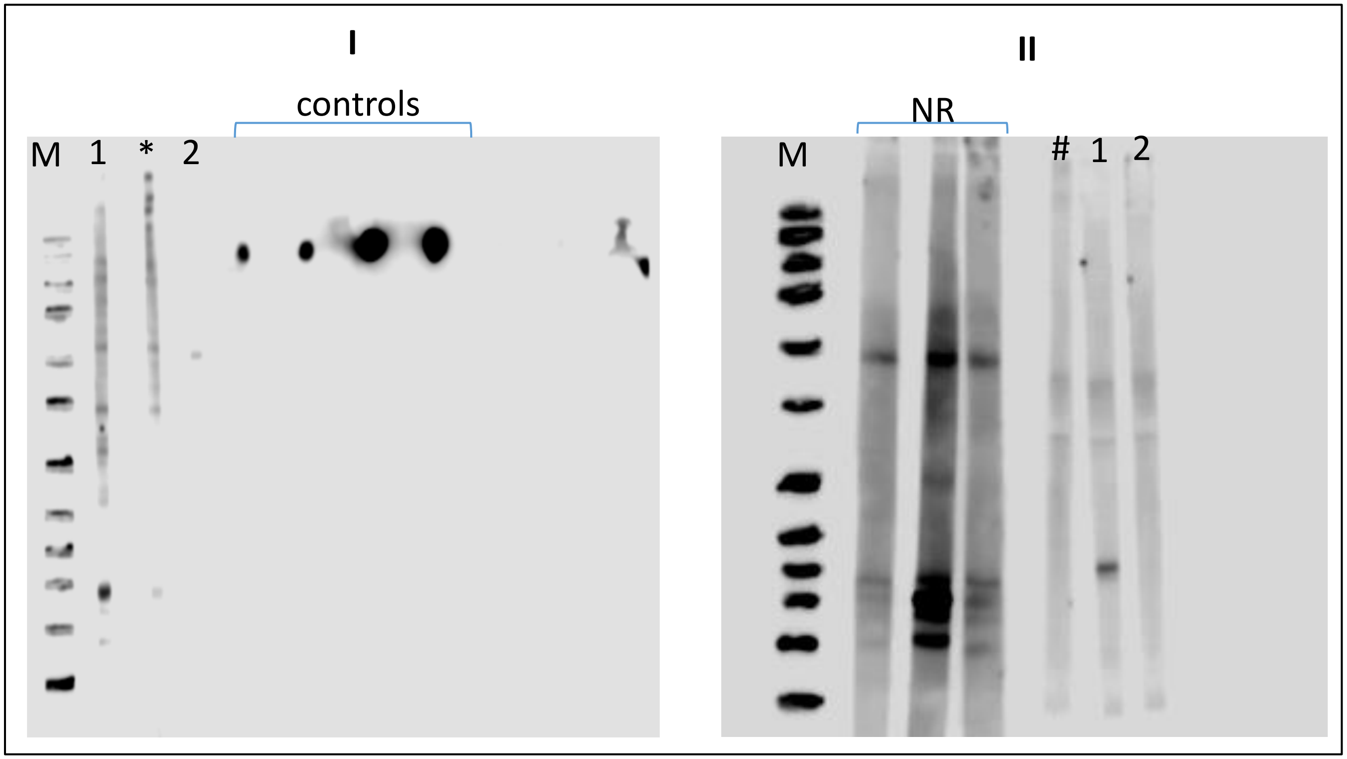

Supplementary Figure S9. Original blots used to make panel A and panel B in Figure 2. *Lanes 1 and 2 in blot I correspond to the lanes 1 and 2 in A of figure 2. Lanes 1 and 2 in blot II correspond to the lanes 1 and 2 in B of figure 2.*

** - ten-fold diluted DIII used in assay (not presented in figure 2). Controls – internal controls of western-blotting (not presented in figure 2) in which control his-tagged protein was spotted on membrane and detected with HisProbe-HRP conjugate and chemiluminescent substrate. # - negative control in duplicate of lane 2 (not presented in figure 2). NR- results not related to this paper.*

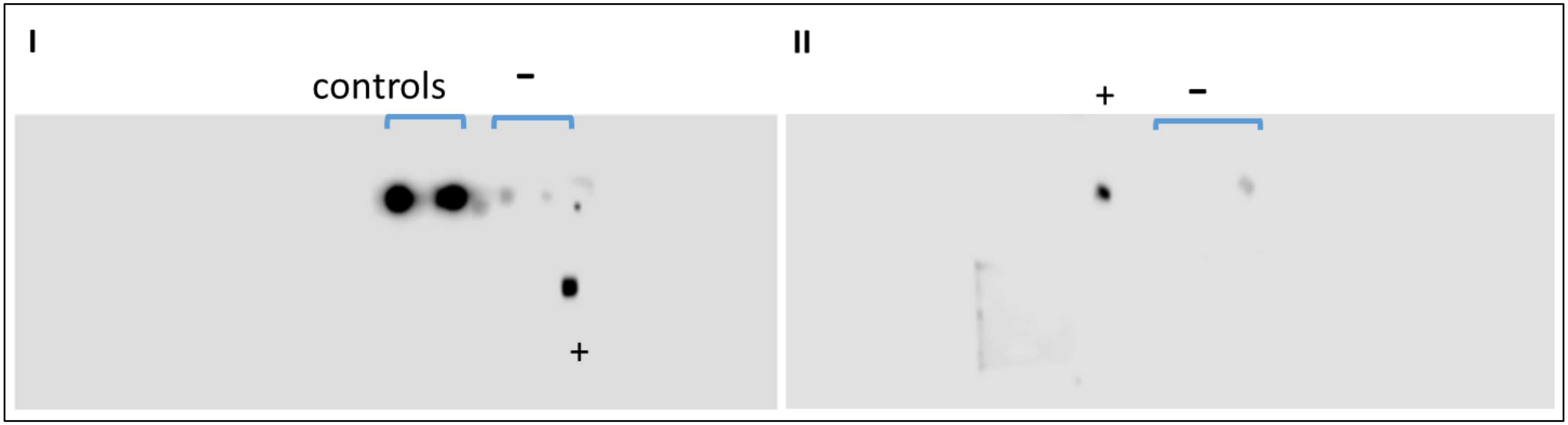

Supplementary Figure S10. Original blots used to make panel D and panel E in Figure 2. *Blot presented above in I was used to make figure 2D. Blot in II was used to make figure 2E. Please note that negative controls in both panels are in duplicate. Controls – duplicate internal controls in western-blotting (not presented in figure 2) in which control his-tagged protein was spotted on membrane and detected with HisProbe-HRP conjugate and chemiluminescent.*

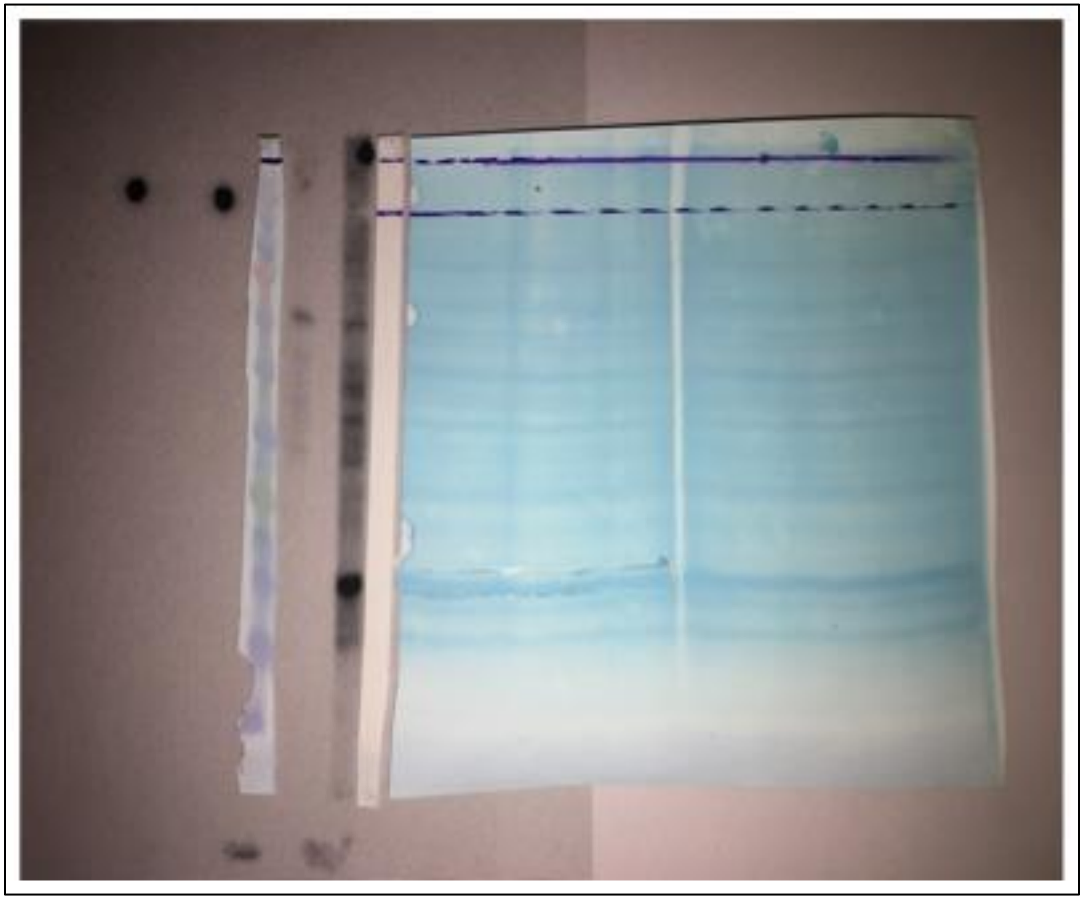

Supplementary Figure S11. Original photo used to make panel C in Figure 2. *Strips were aligned together with NC membrane with transblotted proteins of hBMECs. Alignment was performed to facilitate proper cutting of the prey protein (hBMECs protein interacting with ligands of pathogens).*

SUPPLEMENTARY TABLES

Supplementary Table S1. Amplified sequence of the DIII fragment used for ligation into the pQE-30-mCherry-STOP expression vector for production of recombinant DIII (rDIII)

**nt = 321**	**CTGAAGGGAACAACATATGGA**GTATGTTCAAAAGCGTTTAAATTCCUUGGGACTCCCGCT<br>GACACTGGCCATGGAACGGTGGTGTTGGAACTGCAATACACCGGAACAGACGGTCCCTGC<br>AAAGTGCCCATTTCTTCCGTAGCTTCCCTGAATGACCTCACACCTGTTGGAAGACTGGTGAC<br>CGTGAATCCATTTGTGTCTGTGGCCACAGCCAACGCUAAGGTCTTGATTGAACTCGAACCC<br>CCGTTTGGTGACTCTTACATCGTGGTGGGAAGAGGAGAACAGCAGATAAACCACCAC**TGG**<br>**CACAAATCTGGGAGCAGC**

Sense and antisense primer used for amplification of DIII are indicated in bold.

Supplementary Table S2. Amplified sequence of the NadA fragment used for ligation into the pQE-30-mCherry-STOP expression vector for production of recombinant NadA (rNadA)

**nt = 480**	**ACTTTCTGTAGCGGCGCACTG**GCAGCCACAAGCGACGACGATGTTAAAAAAGCTGCCACT GTGGCCATTGTTGCTGCCTACAACAATGGCCAAGAAATCAACGGTTTCAAAGCTGGAGAG ACCATCTACGACATTGGTGAAGACGGCACAATTACCCAAAAAGACGCAACTGCAGCCGAT GTTGAAGCCGACGACTTTAAAGGTCTGGGTCTGAAAAAAGTCGTGACTAACCTGACCAAAA CCGTCAATGAAAACAAACAAAACGTCGATGCCAAAGTAAAAGCTGCAGAATCTGAAATAG AAAAGTTAACAACCAAGTTAGCAGACACTGATGCCGCTTTAGCAGATACTGATGCCGCTCT GGATGAAACCACCAACGCCTTGAATAAATTGGGAGAAAATATAACGACATTTGCTGAAGA GACTAAGACAAATATCGTAAAAATTGATGAAAAATTA**GAAGCCGTGGCTGATACCGTC**

Sense and antisense primers used for amplification of NadA are indicated in bold.

Supplementary Table S3. Different striping buffers and desalting strategies used to recover bound peptides from the membrane

Name	Stripping/elution buffer used for protein/peptide recovery	Conditions for stripping	Protein/peptide concentration for down-streaming mass spectrometry
harsh stripping buffer (NC membrane)	10% SDS, 0.5 M Tris-HCl, 50 mM DTT, pH 6.8	Strip incubated in pre-warmed stripping buffer at 50°C for 45 min with agitation.	acetone precipitation
glycine-HCl buffer (NC membrane)	50 mM glycine-HCl, pH 2.2	Strip incubated in pre-warmed stripping buffer at 50°C for 45 min.	acetone precipitation
Millipore's stripping buffer – low pH buffer (NC membrane)	25 mM glycine-HCl, and 1% SDS, pH 2.0	Membrane incubated at room temperature in stripping buffer with agitation.	No purification, the pellet was vacuum dried and dissolved with HCCA matrix directly for mass spectrometry.
**Millipore's stripping buffer – low pH buffer (NC membrane)**	**25 mM glycine-HCl, and 1% SDS, pH 2.0**	**Membrane incubated at room temperature for 30 min followed by 15 min at 50°C.**	**acetone precipitation**
low pH buffer with TFA and acetonitrile (PVDF membrane)	25% TFA acid + acetonitrile [1:1 v/v]	Membrane sonicated in the presence of elution buffer for 15 min.	Supernatant was vacuum dried, TA50 was added and desalting with ZipTip® was performed.
**low pH buffer with formic acid and acetonitrile (PVDF membrane)**	**formic acid and acetonitrile [1:5 v/v]**	**Membrane vortexed in the presence of formic acid followed by addition of acetonitrile and sonication for 15 min.**	**The contents were vacuum dried and dissolved in TA50. Next, 0.1% TFA was added and desalting with ZipTip® was performed.**

Conditions with bold are the best among in terms of the recovery of peptides/proteins and thus were used in the experiment

Supplementary Table S4. Buffers used to dissolve synthetic analogues of putative-receptor binding sites (identified in this study)

**Peptide name**	**Mol. Mass of the biotinylated synthetic peptide [Da]**	**Sequence**	**Buffer used to dissolve synthetic peptide**
DIII-1	1142.26	GTTYGVCSK-biotin	phosphate buffer (pH 7.0)
DIII-2	2356.80	VLIELEPPFGDSYIVVGRK-biotin	5 M urea in phosphate buffer (pH 7.0)
NadA-1	4046.90	AATVAIVAAYNNGQEINGFK**AGETIYDIGEDGTITQK**-biotin	4 M urea in phosphate buffer (pH 7.0)
NadA-2	4093.45	LADTDAALADTDAALDETTNALNK**LGENITTFAEETK**-biotin	4 M urea in phosphate buffer (pH 7.0)

In NadA-1 underlined sequence corresponds to the peptide with molecular mass ~2051 Da and bold sequence represents peptide with molecular mass ~1810 Da identified as putative-receptor binding sites of rNadA. These peptides are located one after another in the NadA sequence, therefore combined peptide (NadA-1) was synthetized.
In NadA-2 underlined sequence corresponds to the peptide with molecular mass ~2433 Da and bold sequence represents peptide with molecular mass ~1453 Da identified as putative-receptor binding sites of rNadA. These peptides are located one after another in the NadA sequence, therefore combined peptide (NadA-2) was synthetized.

Supplementary Table S5. Primers used in this study

No.	Protein	Sequence used to desing primers	Primer	Sequence (5´– 3´)	Amplicon lenght (bp)
1	protein E domain III	DQ116961.1*	sense	ACAGGATCCCTGAAGGGAACAACATATGGA	339
			antisense	CTTGTCGACGCTGCTCCCAGATTTGTGCCA	
2	Neisserial adhesion A protein, NadA globular domain	AE002098.2*	sense	AAAGGATCCACTTTCTGTAGCGGCGCACTG	498
			antisense	AAAGGTACCGACGGTATCAGCCACGGCTTC	
3	–	–	UA-INSERTOM-His-sense	CGCATCACCATCACCATCACG	–
			UA-INSERTOM-GFP-antisense	ACCAAATTGGGACAACACCAGTG	

*shows Genbank accession number. Restriction sites are depicted with underlined nucleotides. GGATCC- *BamH*I, GGTACC – *Kpn*I and GTCGAC - *Sal*

SUPPLEMENTARY METHODS

Supplementary Method S1

Human brain microvascular endothelial cell (hBMEC) culture. In short, hBMEC/D3 cell line was obtained from Merck/Millipore (Prague, Czech Republic) and cultured in 75 cm^2 cell culture flask coated with collagen type I (Sigma, USA) in EBM-2 medium (Lonza, UK) containing 10% FBS, gentamycin, 1.4 µM hydrocortisone (Sigma), 5 µg/mL ascorbic acid, 10 mM HEPES and 1 ng/mL bFGF (Sigma). Cells were incubated at 37°C in a humid atmosphere of 5% CO_2 until confluence. Cells from the monolayer (7th passage) were used in the experiment. The confluent monolayer was gently scraped and proteins were isolated under native conditions. In short, cells were harvested by centrifugation (3,000 × g, 10 minutes). Pelleted cells were resuspended in 200 µL of lysis solution (cell surface protein isolation kit, Thermo Fisher Scientific) and incubated 30 minutes on ice (during incubation cells were vortexed for 5 seconds after every 5 minutes). Finally, the cells were disrupted with 5 cycles of sonication on ice (100% amplitude, 30 s). Debris was removed by centrifugation (10,000 × g, 5 minutes, 4 ºC) and protein fraction was subjected for gel filtration (sephadex G-25) against PBS (pH 7.4). Protein concentration was measured with Bradford assay and proteins were stored at -80°C till use.

Supplementary Method S2

Selection and culture of clones for recombinant protein production. The gene fragments encoding domain III (DIII) of the envelope protein of WNV and globular domain of NadA of *N. meningitidis* were amplified by PCR from genomic DNA. The amplified fragment was digested with restriction enzymes *BamH*I and *Sal*I or *Kpn*I (Thermo Fisher Scientific, Slovakia), as per the

manufacturer's instruction. Digested fragments were ligated into a pQE-30-mCherry-STOP plasmid **(Supplementary Figure S2)**. Please note that in this vector mCherry serves as a stuffer sequence, which is cut out during the digestion of vector with restriction enzymes. Ligation mix contained 2 µl of ligation buffer, 2 µl of PEG 8000, 0.5 U of the T4 ligase, amplicon and plasmid (50 ng) in molar ratio of 10:1, and water up to 20 µl. Ligation was performed at 22°C for 1 h. Ligation mix was purified using NucleoSpin (Macherey-Nagel, Germany) and transformed into *E. coli* M15 strain (Qiagen, Germany). Transformants were selected from LB agar plates (lysogeny broth, Sigma; supplemented with 2% bacteriological agar, 1% glucose, 25 µg/mL kanamycin and 50 µg/mL carbenicillin). Presence of encoding gene in transformants was confirmed by sequencing (vector-specific primers UA Insertom F and R, presented in **Supplementary Table S5**).

A single colony carrying insert gene was cultivated in Terrific broth (TB) (15 g/L tryptone, 30 g/L yeast extract, 12.5 g/L NaCl, 2.5 g/L $MgCl_2/MgSO_4$, 100 µL/L metal mix, 7.5 mL/L glycerol) supplemented with 1% glucose, 50 µg/mL carbenicillin and 25 µg/mL kanamycin until $OD_{600} = 6$. Bacterial cells were pelleted (centrifugation at 6,000 × *g* for 10 min) and resuspended in fresh TB medium without glucose. Protein expression was induced with 1 mM IPTG (Fermentas, Slovakia) at 30°C for 8 h for rDIII and 20°C for 18 h for rNadA.

Supplementary Method S3

Isolation and purification of recombinant proteins. After induction, cells were pelleted (17,880 × *g* for 10 min) and lysed in lysis buffer (0.03 M Na_2HPO_4, 0.5 M NaCl, 0.001% Tween® 20, 10% glycerol, 1x protease inhibitor cocktail, Sigma) with four freeze-thaw cycles followed by sonication on ice (2 cycles; 30-s pulses, 100% amplitude). Proteins were purified with nickel affinity

chromatography (Ni-NTA agarose beads, ABT agarose, Spain) as per manufacturer´s instructions. Briefly, Ni-NTA beads were equilibrated with the buffer (50 mM Na_2HPO_4, 300 mM NaCl, 0.001% Tween® 20, pH 8.0). Beads were then incubated in the lysate for 2 h at 8°C with constant rotation (140 rpm). Unbound proteins were washed with washing buffer (50 mM Na_2HPO_4, 300 mM NaCl, 0.001% Tween® 20, 20 mM imidazole, pH 8.0) for 5 times and protein were eluted in elution buffer (50 mM Na_2HPO_4, 300 mM NaCl, 250 mM imidazole, pH 8.0). Proteins were immediately subjected to gel filtration (Sephadex G25, 30 ml column, *in-house* prepared) on ÄKTApurifier (GE-Healthcare, 2 ml/min flow, max 0.45 MPa pressure) to remove imidazole and accomplish the buffer exchange.

In the case of rNadA, the buffer used for exchange was Bis-Tris, pH 6.0 containing 8M urea. Protein was immediately processed with anion exchange chromatography (polishing step) on ÄKTApurifier with following conditions: column – Resource Mono Q (1 ml, GE Healthcare), start buffer - Bis-Tris, pH 6.0 containing 8 M urea, elution buffer - Bis-Tris, pH 6.0 with 1 M NaCl, pH 6.0, flow rate – 1 ml/min, gradient 0% to 100% of elution buffer in 5 CV (column volume). Please note that scouting was performed to standardize elution conditions described above (scouting of various gradient, salt concentration, and CVs). The gradient of 5 CV was kept finally to concentrate the proteins. Subsequent dialysis against PBS was performed to remove NaCl.

The purity of recombinant domains was re-evaluated with LDS-PAGE and molecular weight was confirmed by MALDI-TOF MS. Aliquots of purified rDIII and rNadA were stored in 10% glycerol at - 20°C until further use.

Supplementary Method S4

LDS-PAGE and MALDI-TOF

Whole-cell lysate of *E. coli*, flow-through from nickel affinity chromatography, recombinant proteins eluted from nickel affinity chromatography, proteins after gel filtration or ion exchange were analyzed by LDS-PAGE. Briefly, the protein samples (20 µl each) were mixed with lithium dodecyl sulfate sample buffer (4X LDS sample buffer, Invitrogen, Slovakia) as per manufacturer's instructions and incubated at 72°C for 10 min. Electrophoresis was carried out at 30 mA in 1X running buffer (20X NuPAGE MOPS SDS running buffer, Invitrogen) until the dye reached the bottom of the gel (12% Bis-Tris polyacrylamide 12 well gel, Invitrogen). Proteins were stained with Coomassie staining (Bio-Rad).

For MALDI-TOF MS, 0.8 µl of the purified proteins were mixed with 0.8 µl sDHB matrix (2,5-2,5-dihydroxybenzoic acid and 2-hydroxy-5-methoxybenzoic acid, Bruker Daltonics, USA) dissolved up to saturation in TA50 (50:50 [v/v] acetonitrile:0.1% trifluoroacetic acid, TFA, Sigma). One microliter of the protein-matrix mix was spotted on the ground-steel plate (Bruker Daltonics) and allowed to air dry. The acquisition was performed in flexControl (V 3.4, Burker-Daltonics) in linear mode with 60 Hz laser intensity (200 shots) on Microflex with reflectron MALDI mass spectrometer (Bruker Daltonics). Mass was analyzed in flexAnalysis V3.4 software of Bruker Daltonics by comparing it with calibrants (protein calibration kit I, Bruker-Daltonics).

Supplementary Method S5

Interaction of recombinant ligands and the receptor of hBMECs by ELISA

Protein extract of hBMECs diluted in coating buffer (0.5 M Na_2CO_3, 0.5 M $NaHCO_3$, pH 9.5) was incubated in the microtiter wells (4 µg/well) of ELISA plate for overnight at 4°C. Unbound hBMECs proteins were washed with PBS (pH 7.4) containing 0.05% Tween® 20 (PBST-20) and blocking of non-specific binding sites was performed with 0.5% BSA in PBST-20. Two micrograms of recombinant ligands (rDIII and rNadA, diluted in PBST-20) were added in separate wells and incubated for 3 h at room temperature with constant shaking. Wells were washed with PBST-20 and incubated with HisProbe-HRP conjugate dissolved in PBST-20 (1:5000 dilution, Thermo Fisher Scientific, Slovakia) for 15 min at room temperature. After the rigorous washings, the chromogenic reaction was developed using 1-Step™ Ultra TMB-ELISA substrate (Thermo Fisher Scientific). After 25 min, the reaction was stopped with 2 M H_2SO_4 and absorbance was measured at 450 nm. As an input control, rDIII or rNadA were coated in microtiter wells and HisProbe-HRP conjugate was used for detection. For negative controls, either hBMECs proteins or recombinant ligands were excluded from the experiment. As a blank control, both hBMECs proteins and recombinant ligands were excluded from the experiment. The assay was performed in triplicates.

Supplementary Method S6

Interaction of recombinant ligands and the receptor of hBMECs by Western blotting

Two hundred micrograms of hBMECs proteins separated on 2 well PAGE gel (12% polyacrylamide) were transferred on 0.45 µm NC membrane (Amersham™ Protran®, GE Healthcare, USA) for 1 h. Details of LDS-PAGE are described in **Supplementary Methods S4**. The

transblotted NC was sliced to obtain 0.3 cm vertical strips and used for Western blotting. Non-specific binding sites were blocked with 1% polyvinylpyrrolidone 40000 (PVP-40, Sigma, USA) in TBS (pH 7.2) followed by a washing step with TBS containing 0.05% Tween® 20 (TBST-20). Further on, strips were incubated with 5 µg of purified rDIII or rNadA for 3 h at room temperature. After 3 washings with TBST-20, strips were incubated in HisProbe-HRP conjugate (1:5000 dilution) dissolved in 5 ml of 1% PVP-40 in TBST-20 for 30 min at room temperature. Six washings were performed before incubating the strips in SuperSignal™ chemiluminescent substrate (Thermo Fisher Scientific). The signal was captured on C-DiGit® Blot Scanner (Odyssey CLx, LI-COR Biosciences, UK). In case of negative controls, incubation of the NC strips with recombinant ligands were excluded from the above-mentioned protocol.

Supplementary Method S7

Confirmation of interaction between putative receptor-binding sites and proteins of hBMECs

Putative receptor-binding sites identified above were synthesized commercially (Caslo, Denmark). Peptides were biotinylated in C-terminal lysine. Peptides were dissolved according to the manufacturer's instructions **(Supplementary Table S4)**. ELISA assay was employed to corroborate interaction between peptides and proteins of hBMECs. In short, hBMECs proteins were coated to microtiter wells and non-specific binding sites were blocked as described above. Two micrograms of each peptide were incubated with coated proteins of hBMECs for 3 h at room temperature. After 3 washing with PBST-20, Streptavidin-HRP conjugate (1:10 000, GE Healthcare) was added into the wells for 1 h at room temperature. Wells were washed 6 times with PBST-20 and TMB substrate was added. After 25 min, the reaction was stopped with 2 M

H_2SO_4 and absorbance was measured at 450 nm. The assay was performed in triplicates. For negative controls, either peptides or hBMECs proteins were excluded from the assay. For blank control, only coating buffer and Streptavidin-HRP conjugate were included in the experiment.
